# Antiretroviral Postexposure Prophylaxis After Sexual, Injection Drug Use, or Other Nonoccupational Exposure to HIV — CDC Recommendations, United States, 2025

**DOI:** 10.15585/mmwr.rr7401a1

**Published:** 2025-05-08

**Authors:** Mary R. Tanner, Jesse G. O’Shea, Katrina M. Byrd, Marie Johnston, Gema G. Dumitru, John N. Le, Allison Lale, Kathy K. Byrd, Preetam Cholli, Emiko Kamitani, Weiming Zhu, Karen W. Hoover, Athena P. Kourtis

**Affiliations:** ^1^National Center for HIV, Viral Hepatitis, STD, and TB Prevention, CDC; ^2^U.S. Public Health Service Commissioned Corps

## Abstract

Nonoccupational postexposure prophylaxis (nPEP) for HIV is recommended when a nonoccupational (e.g., sexual, needle, or other) exposure to nonintact skin or mucous membranes that presents a substantial risk for HIV transmission has occurred, and the source has HIV without sustained viral suppression or their viral suppression information is not known. A rapid HIV test (also referred to as point-of-care) or laboratory-based antigen/antibody combination HIV test is recommended before nPEP initiation. Health care professionals should ensure the first dose of nPEP is provided as soon as possible, and ideally within 24 hours, but no later than 72 hours after exposure. The initial nPEP dose should not be delayed due to pending results of any laboratory-based testing, and the recommended length of nPEP course is 28 days.

* The recommendations in these guidelines update the 2016 nPEP guidelines (*CDC. Updated guidelines for antiretroviral postexposure prophylaxis after sexual, injection drug use, or other nonoccupational exposure to HIV — United States, 2016. Atlanta, GA: US Department of Health and Human Services, CDC; 2017*). These 2025 nPEP guidelines update recommendations and considerations for use of HIV nPEP in the United States to include newer antiretroviral (ARV) agents, updated nPEP indication considerations, and emerging nPEP implementation strategies. The guidelines also include considerations for testing and nPEP regimens for persons exposed who have received long-acting injectable ARVs in the past. Lastly, testing recommendations for persons who experienced sexual assault were updated to align with the most recent CDC sexually transmitted infection treatment guidelines.*

These guidelines are divided into two sections: Recommendations and CDC Guidance. The preferred regimens for most adults and adolescents are now bictegravir/emtricitabine/tenofovir alafenamide or dolutegravir plus (tenofovir alafenamide or tenofovir disoproxil fumarate) plus (emtricitabine or lamivudine). However, the regimen can be tailored to the clinical circumstances. Medical follow-up for persons prescribed nPEP also should be tailored to the clinical situation; recommended follow-up includes a visit at 24 hours (remote or in person) with a medical provider, and clinical follow-up 4–6 weeks and 12 weeks after exposure for laboratory testing. Persons initiating nPEP should be informed that pre-exposure prophylaxis for HIV (PrEP) can reduce their risk for acquiring HIV if they will have repeat or continuing exposure to HIV after the end of the nPEP course. Health care professionals should offer PrEP options to persons with ongoing indications for PrEP and create an nPEP-to-PrEP transition plan for persons who accept PrEP.

## Introduction

With advances in HIV prevention and care efforts in the United States, estimated new HIV infections have declined from a peak of 130,000 annually in the mid-1980s to 32,800 in 2022 (*1*). Further progress in preventing new infections is critical to ending the HIV epidemic. One tool to prevent HIV infection is HIV postexposure prophylaxis (PEP). This effective intervention uses antiretroviral (ARV) medications to reduce the likelihood of HIV acquisition after high-risk exposures. PEP can be nonoccupational (nPEP, e.g., after sexual, needle, or other exposure) or occupational (oPEP, e.g., after needlestick injury during surgery) (*2*). Although the medications prescribed for nPEP and oPEP are identical, populations of nPEP and oPEP users differ substantially. For instance, oPEP users are typically health care providers or first responders, whereas nPEP users are persons who were exposed to HIV during sexual or injection drug use behavior. As a result, special considerations are needed for the care of nPEP populations and are addressed in these guidelines.

The U.S. Department of Health and Human Services (HHS) first provided nPEP recommendations in 2005, which were updated in 2016 to include newer ARV regimens, their side-effect profiles, and cost-effectiveness of nPEP to prevent HIV infection for different exposure types (*3*). These 2025 nPEP guidelines update recommendations and considerations for use of HIV nPEP in the United States to include newer ARV agents, updated nPEP indication considerations (e.g., for persons taking HIV pre-exposure prophylaxis [PrEP] and exposed to HIV), and emerging nPEP implementation strategies. This update also includes considerations for testing and nPEP regimens for persons exposed who have received long-acting injectable ARVs in the past. Lastly, testing recommendations for persons who experienced sexual assault were updated to align with the most recent CDC sexually transmitted infection (STI) treatment guidelines (*4*). A detailed list of updates is presented (Box 1). These recommendations are intended to guide U.S. health care professionals’ clinical management of adults and children potentially exposed to HIV outside of occupational settings.

BOX 1 **What’s new in the guidelines — CDC recommendations for HIV nonoccupational postexposure prophylaxis, United States, 2025**

This update incorporates new evidence published since the 2016 HIV nonoccupational postexposure prophylaxis (nPEP) guidelines, including evidence related to safety and tolerability of newer antiretroviral (ARV) medications. Highlighted updates from the 2016 guideline recommendations include the following:

- Expanded discussion of nPEP indications, now including situations in which the source (e.g., a person with HIV) has consistent viral suppression, in which the person exposed has been taking HIV pre-exposure prophylaxis (PrEP), and in which the known or possible exposure occurred due to sexual assault (see HIV nPEP Indications).
- Increased emphasis on the urgency of nPEP initiation including optimal administration within the first 24 hours after exposure (see Time to Initiation of HIV nPEP).
- The preferred regimens for most adults and adolescents are now bictegravir (BIC)/emtricitabine (FTC)/tenofovir alafenamide (TAF) OR dolutegravir (DTG) plus tenofovir alafenamide (TAF) OR tenofovir disoproxil fumarate (TDF) plus emtricitabine (FTC) OR lamivudine (3TC). Guidance about preferred and alternative regimens for adults and adolescents, children, and pregnant women, and regimens for persons with renal or hepatic dysfunction also is provided (see HIV nPEP Regimens).
- Updated clinical considerations for laboratory testing including considerations for use of diagnostic HIV nucleic acid tests (NATs) along with HIV antigen/antibody tests (Ag/Ab tests) in certain clinical scenarios; emphasis on not delaying nPEP initiation while awaiting laboratory testing; update to routine follow-up testing of serum creatinine, alanine aminotransferase (ALT), and aspartate aminotransferase (AST), indicating that it is not necessary unless baseline tests are abnormal or other specific clinical indications are present; and updates to testing for other clinical conditions such as other sexually transmitted infections (STIs) (see Laboratory Testing and nPEP Follow-Up).
- Emphasis on the need for HIV PrEP education for all persons assessed for nPEP, and the need for an nPEP-to-PrEP transition plan when indicated, including the possibility of an immediate PEP-to-PrEP transition for persons with substantial likelihood of HIV acquisition who might benefit from this approach (see Transitioning to PrEP after PEP).

The National Clinician Consultation Center (NCCC) provides tailored nPEP clinical consultation for U.S. health care professionals (888-448-4911 or https://nccc.ucsf.edu/clinician-consultation/pep-post-exposure-prophylaxis). Separate guidelines as well as consultation services with NCCC are available for occupational HIV PEP (oPEP) (*2*); HIV PrEP (*5*), which is the use of ARVs before HIV exposure to reduce the likelihood of HIV acquisition; and prevention of perinatal HIV transmission (*6*).

HIV nPEP guideline development is challenging because of limitations in the published literature. Few clinical trial data are available, and most of these data are related to safety and tolerability of ARVs when used as nPEP. Historically, nPEP recommendations have been based on observational data, animal models and extrapolated data from HIV treatment, prevention of perinatal HIV transmission, PrEP, and oPEP studies as well as expert opinion. These 2025 recommendations are based on clinical experience, subject matter expertise, and data published since 2016 on the established clinical practice of nPEP. As a result of these limitations, there are no Food and Drug Administration (FDA)-approved medications specifically for nPEP. Therefore, all listed medications recommended for nPEP represent off-label use.

## Methods

The recommendations in these guidelines update the *Updated Guidelines for Antiretroviral Postexposure Prophylaxis After Sexual, Injection Drug Use, or Other Nonoccupational Exposure to HIV — United States, 2016* (hereafter referred to as the 2016 nPEP guidelines) (*2*). In December 2021, CDC assembled a work group of agency subject matter experts who identified the priority topic areas for the update of these guidelines (hereafter referred to as the work group). Separately, in February 2022, CDC reconvened the interagency U.S. Public Health Service (PHS) work group to plan and prepare an update of the* Updated U.S. Public Health Service (PHS) Guidelines for the Management of Occupational Exposures to Human Immunodeficiency Virus and Recommendations for Postexposure Prophylaxis, 2013* (hereafter referred to as the 2013 PHS guidelines) (*4*). The two work groups conducted literature reviews, which included systematic literature reviews according to Preferred Reporting Items for Systematic Reviews and Meta-Analyses (PRISMA) reporting guidelines (*7*) and included both occupational and nonoccupational exposures (*8*). The HIV nPEP guidelines update process proceeded with the categorization of evidence, evaluation of evidence quality, development of recommendations, external input, and peer review.

### Literature Search and Evidence Sources

The 2016 nPEP guidelines included evidence identified through a search of the scientific literature published from January 2005 to July 2015 (*2*). The 2025 update adds evidence published from January 2015 to January 2024. A CDC librarian assisted in developing the search strategy. A systematic literature search was performed in Medline, Embase, PsycINFO, Cochrane Library, CINAHL, and Scopus databases without language restrictions. Search terms included “HIV post exposure prophylaxis,” “postexposure prophylaxis,” “nPEP,” “nonoccupational postexposure or post-exposure prophylaxis,” “HIV postexposure or post-exposure prophylaxis,” “post exposure or postexposure prevention,” “nonoccupational,” “non-PEP (nonoccupational postexposure prophylaxis),” “NOPEP (nonoccupational postexposure prophylaxis),” “PEP (postexposure prophylaxis),” “post-exposure prophylaxis after sexual exposure,” and “self-start HIV postexposure prophylaxis (PEPSE).” Duplicates were identified using the Endnote automated “find duplicates” function with preferences set to match on title, author, year, and removed. Additional deduplication occurred during the review and categorization process.

### Study Selection and Categorization

CDC completed the screening and full-text review of the literature in Covidence, a web-based systematic review software, in November 2024 (*9*). Two reviewers independently screened titles and abstracts; differences in inclusion or categorization were resolved by a third reviewer. Included studies went on to full-text article review. Studies were included based on the nPEP inclusion and exclusion criteria. The inclusion criteria for both the titles and abstracts screening and full-text review included scientific publications, English language, human data, data relevant to HIV nPEP (e.g., nPEP interventions, HIV acquisition, and ARV regimen completion), and literature published in peer-reviewed journals or in *Morbidity and Mortality Weekly Report*. The exclusion criteria for the screening were as follows: non-English language; non-scientific article; does not contain HIV and a version of postexposure prophylaxis in the title or abstract; and non-human studies. Exclusion criteria for the full-text review were as follows: studies outside the United States unless they contained data on ARVs used for nPEP, adherence outcomes, or adverse effects; nPEP studies in non-human models; commentary or otherwise non–peer-reviewed study; study of nPEP epidemiology before 2018; and publication withdrawn or otherwise inaccessible.

**FIGURE 1:**
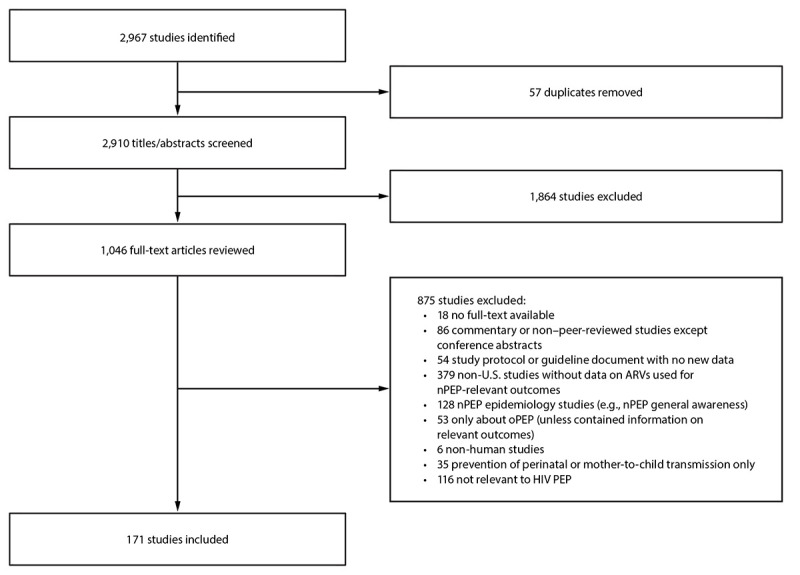
Study selection process for updating HIV nonoccupational postexposure prophylaxis recommendations — CDC recommendations, United States, 2025 **Abbreviations:** ARV = antiretroviral; nPEP = nonoccupational postexposure prophylaxis; oPEP = occupational postexposure prophylaxis; PEP = postexposure prophylaxis.

The results of the study selection process are depicted (Figure 1)*.* The nPEP guideline update team categorized the 171 studies meeting the inclusion criteria into topic areas, which were then presented to and considered by the nPEP guideline update work group. Topic areas included health care access or system related to nPEP, nPEP window period and duration, nPEP awareness and use by health care professionals, nPEP awareness and use by clients, nPEP-to-PrEP transition, nPEP-in-pocket approach, nPEP regimens recommended in 2016 guidelines, new possible nPEP regimens, and nPEP and HIV testing.

### Evaluation of Evidence Quality and Development of Recommendations

The nPEP guidelines update team defined key outcomes relevant to the nPEP topic areas. New evidence-based recommendations were developed using the Grading of Recommendations, Assessment, Development, and Evaluation (GRADE) framework (*10*). The GRADE framework also was applied to all key questions in the systematic review. Recommendations that were not informed by systematic review were classified as good practice statements according to the criteria set forth by GRADE (*11*). Recommendations and good practice statements were of equal importance; however, they reflected different underlying methodologic processes. For these recommendations, CDC weighed the overall certainty of the evidence, the balance of benefits and harms, users’ values and preferences, acceptability, feasibility, and equity per GRADE methodology. In this update, the classification as a good practice statement includes existing recommendations brought forward from CDC guidelines and new recommendations informed by indirect pharmacokinetic or mechanism of action data or practices determined to be standard of care based on the expert experience of the work group or individually consulted external subject matter experts (*2*,*5*,*12*) (Supplementary Appendix A: Recommendation Strength & Rationale, https://stacks.cdc.gov/view/cdc/177225#tabs-3). Studies underlying the good practice statements were not amenable for rating the certainty of evidence.

The GRADE process provided a systematic evaluation of all the evidence and quality that contributed to the work group’s understanding of the topic areas and subsequent selection of recommendation ratings. The Newcastle-Ottawa scale was applied to each study to assess bias (*13*). The GRADE rating tables are available (Supplementary Appendix B: GRADE Tables, https://stacks.cdc.gov/view/cdc/177225#tabs-3). Most recommendations were agreed by entire work group consensus; all recommendations were approved by at least a two thirds majority vote (*14*). All draft recommendations were shared with the PHS work group to harmonize recommendations between guidelines where applicable.

### Review Process Including External Input, Peer Review, and CDC Clearance

Draft recommendations were presented to health care professionals through two listening sessions (webinars) organized with the Infectious Diseases Society of America and the HIV Medicine Association. These sessions were attended by approximately 100 participants who shared individual input only; no group consensus was sought. All feedback was reviewed, organized topically, and considered in draft revisions. The draft guideline document was also submitted for external peer review by three reviewers who were experts in HIV prevention and nPEP, through the Office of Management and Budget’s process, and this input guided draft revisions (*15*). Feedback was synthesized, prioritized, and incorporated, as necessary, by CDC, and the finalized draft guidelines were entered into CDC clearance in May 2024.

All authors, contributors, and providers of external input were asked to disclose any active potential conflicts of interest related to the 2025 nPEP guideline update (Supplementary Appendix C: 2024 nPEP Guideline Update Authors, Contributors, and Conflicts of Interest, https://stacks.cdc.gov/view/cdc/177225#tabs-3). The work group reported associations for potential competing interests and determined the appropriate action, as follows: disqualification from the work group, disqualification or recusal from topic review and discussion, or no disqualification needed. An active competing interest was defined as any current, direct financial interest related to a product addressed in the section of the guideline to which the person contributed content. Financial interests included direct receipt of payments, gratuities, consultancies, honoraria, employment, grants, support for travel or accommodation, or gifts from an entity having a commercial interest in that product. Financial interest also included direct compensation for membership on an advisory board, data safety monitoring board, or speakers’ bureau. Compensation and support awarded to an employing university or institution (e.g., grants or research funding) were not considered a competing interest.

### Plans for Guideline Updates

CDC plans to conduct systematic literature reviews regularly and update the guidelines as indicated when new information emerges in the field. These updates are planned at regular intervals, or if substantial changes in practices or new evidence indicate that an update to the recommendations is needed. These guidelines strive to recommend optimal HIV nPEP delivery, and the emergence of new data in the following areas might prompt an update: 1) nPEP time to initiation, 2) nPEP duration, 3) 2-drug nPEP regimens, and 4) long-acting injectable ARVs for nPEP.

## Recommendations

The following section provides recommendations to clinicians from the beginning of the nPEP evaluation to the completion of nPEP therapy (Box 2). “HIV nPEP Indications” details the situations in which nPEP should and should not be administered. “Time to Initiation of HIV nPEP” stresses rapid initiation of nPEP and explains the rationale behind this recommendation. “HIV nPEP Regimens” provides preferred and alternative nPEP regimens. “Laboratory Testing and nPEP Follow-Up” outlines initial and monitoring laboratory tests recommended for nPEP. Finally, “Transitioning to PrEP after PEP” outlines the indications for and management of transitioning persons from nPEP to PrEP.

BOX 2 **CDC recommendations for HIV nonoccupational postexposure prophylaxis, United States, 2025**

**HIV nPEP Indications**

- nPEP is recommended when an exposure has occurred within the past 72 hours that presents a substantial risk for HIV transmission and the source has HIV without sustained viral suppression or their viral suppression information is not known (**good practice statement, existing recommendation**).
- A case-by-case determination is required when an exposure has occurred within the past 72 hours that presents a substantial risk for HIV transmission, but it is not known whether the source has HIV (**good practice statement, existing recommendation**).
- nPEP is not recommended if the exposure presents no substantial risk for HIV transmission (**good practice statement, existing recommendation**).
- nPEP should be stopped if at any point during the course the source is found to not have HIV (**good practice statement, existing recommendation**).

**Time to Initiation of HIV nPEP**

- Initiate nPEP as soon as possible, but no later than 72 hours after exposure (NEW*: **good practice statement, existing recommendation**).

**HIV nPEP Regimens**

- Complete a clinical assessment before prescribing nPEP, including assessing for medical comorbidities, current medications, and allergies (**good practice statement, standard of care**).
- The recommended nPEP course is 28 days (**good practice statement, existing recommendation**).
- The preferred regimens for adults and adolescents without relevant contraindications are
  º bictegravir (BIC)/emtricitabine (FTC)/tenofovir alafenamide (TAF) (NEW*: **recommendation, very low certainty of evidence**) OR
  º dolutegravir (DTG) plus (tenofovir alafenamide [TAF]) OR tenofovir disoproxil fumarate [TDF]) plus (emtricitabine [FTC] OR lamivudine [3TC]) (NEW*: **recommendation, very low certainty of evidence**).
- Selection of a regimen should be individualized based on comorbid conditions (e.g., renal or hepatic dysfunction), pregnancy, drug interaction potential with concurrent medications, previous exposure to ARV regimens (including long-acting injectable ARV exposure), the source’s history, and regimen factors that might influence continuation of treatment (e.g., pill burden, dosing frequency, side effects, cost, and access) (**good practice statement, standard of care**).

**Laboratory Testing and nPEP Follow-Up**

- Persons being assessed due to a known or possible exposure to HIV should be tested for HIV (**good practice statement, existing recommendation**).
- At the initial nPEP medical visit, a rapid (also referred to as point-of-care), laboratory-based antigen/antibody combination (Ag/Ab) HIV test, or both, is recommended (**good practice statement, existing recommendation**).
- For persons with long-acting injectable PrEP ARV exposure during the past 6 months, a diagnostic HIV nucleic acid test (NAT) is recommended at the initial medical evaluation, in addition to an Ag/Ab HIV test (NEW*: **good practice statement, indirect data; existing recommendation**).
- Perform interim HIV testing with both a laboratory-based HIV Ag/Ab test plus a diagnostic HIV NAT test 4–6 weeks after exposure (**good practice statement, standard of care**).
  º HIV testing 4–6 weeks post-nPEP initiation may be deferred for persons who started nPEP within 24 hours of a known or possible HIV exposure and who did not miss any nPEP doses.
- Perform final HIV tests using laboratory-based HIV Ag/Ab combination immunoassay and diagnostic HIV NAT 12 weeks after exposure (NEW*: **good practice statement, standard of care**).
- Routine laboratory testing recommended for persons starting nPEP includes serum creatinine, alanine aminotransferase (ALT), and aspartate aminotransferase (AST), as well as HIV, hepatitis B virus (HBV), and pregnancy testing (**good practice statement, existing recommendation**).
- Testing and treatment of hepatitis C virus (HCV) infection, other sexually transmitted infections including gonorrhea, chlamydia, and syphilis, and other medical treatment should be tailored to the clinical situation (**good practice statement, existing recommendation**).

**Transitioning to PrEP After PEP**

- An immediate transition from nPEP to PrEP, including HIV testing at the completion of the nPEP regimen with a prompt transition to a recommended PrEP regimen, might be beneficial for persons with anticipated repeat or ongoing potential HIV exposures (**good practice statement, existing recommendation**).

* NEW indicates a new recommendation or an update to an existing recommendation.

### HIV nPEP Indications

HIV nPEP is indicated to reduce the risk for acquiring HIV from an exposure that presents a substantial risk for HIV acquisition. Assessing the likelihood of HIV acquisition associated with an exposure requires consideration of multiple factors, including whether the source has HIV, the source’s level of viremia, the body fluid involved in the exposure, the exposure site, presence of barriers to body fluid exposure, and whether the exposed person is on PrEP.

#### Recommendations for HIV nPEP Indications

- nPEP is recommended when an exposure has occurred within the past 72 hours that presents a substantial risk for HIV transmission and the source has HIV without sustained viral suppression or their viral suppression information is not known (**good practice statement, existing recommendation**).
- A case-by-case determination is required when an exposure has occurred within the past 72 hours that presents a substantial risk for HIV transmission, but it is not known whether the source has HIV (**good practice statement, existing recommendation**).
- nPEP is not recommended if the exposure presents no substantial risk for HIV transmission (**good practice statement, existing recommendation**).
- nPEP should be stopped if at any point during the course the source is found to not have HIV (**good practice statement, existing recommendation**).

#### Rationale for HIV nPEP Indications

**FIGURE 2:**
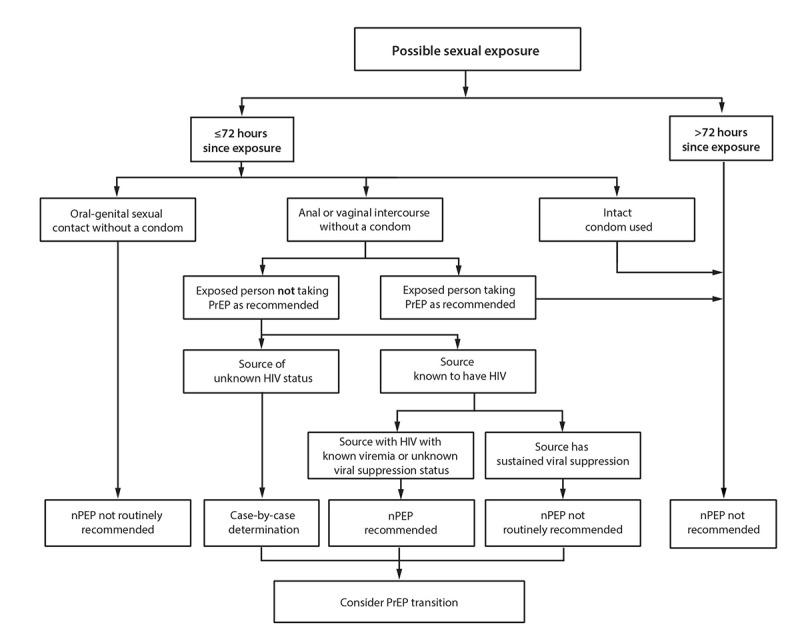
HIV nonoccupational postexposure prophylaxis in the setting of possible sexual exposure — CDC recommendations, United States, 2025*^,†^ **Abbreviations:** nPEP = nonoccupational postexposure prophylaxis; PrEP = pre-exposure prophylaxis. * Evidence is insufficient to recommend nPEP initiation later than 72 hours postexposure. However, certain experts have argued that risk versus benefit considerations could favor a longer initiation window. ^†^ See Appendix A for more information on case-by-case determinations. Health care professionals unfamiliar with nPEP should use local infectious diseases or other expert consultation resources or consult the National Clinical Consultation Center PEPline at 888-448-4911 or https://nccc.ucsf.edu/clinician-consultation/pep-post-exposure-prophylaxis, or the Perinatal HIV Line at 888-448-8765 or https://nccc.ucsf.edu/clinician-consultation/perinatal-hiv-aids.

**FIGURE 3:**
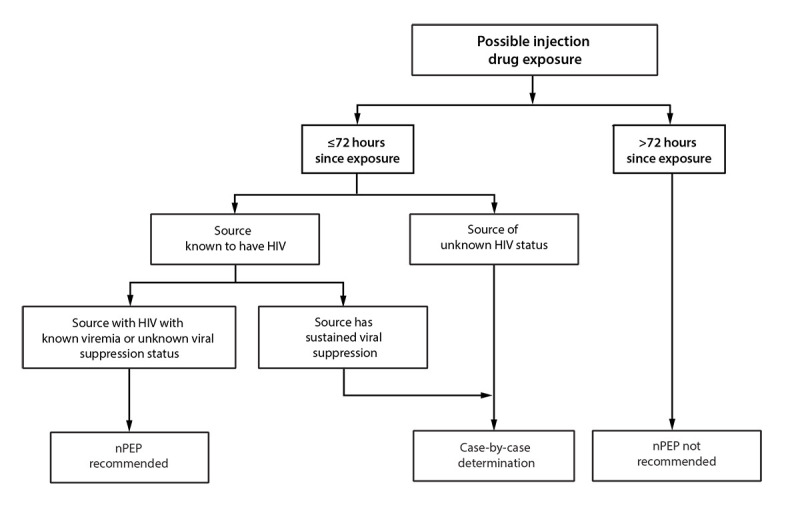
HIV nonoccupational postexposure prophylaxis in the setting of possible injection drug use — CDC recommendations, United States, 2025* **Abbreviation:** nPEP = nonoccupational postexposure prophylaxis. * See Appendix A for more information on case-by-case determinations. Health care professionals unfamiliar with nPEP should use local infectious diseases or other expert consultation resources or consult the National Clinical Consultation Center PEPline at 888-448-4911 or https://nccc.ucsf.edu/clinician-consultation/pep-post-exposure-prophylaxis, or the Perinatal HIV Line at 888-448-8765 or https://nccc.ucsf.edu/clinician-consultation/perinatal-hiv-aids.

**FIGURE 4:**
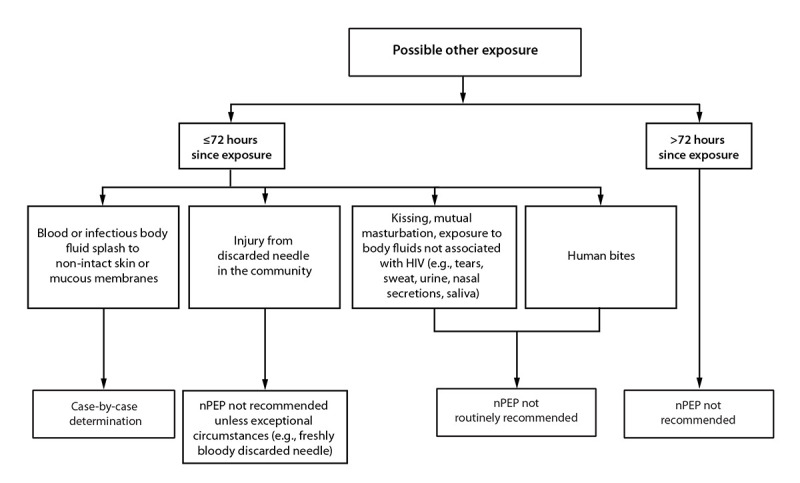
HIV nonoccupational postexposure prophylaxis in the setting of infective fluid splash or exposure, needle injury, or human bites — CDC recommendations, United States, 2025* **Abbreviation:** nPEP= nonoccupational postexposure prophylaxis. * See Appendix A for more information on case-by-case determinations. Health care professionals unfamiliar with nPEP should use local infectious diseases or other expert consultation resources or consult the National Clinical Consultation Center PEPline at 888-448-4911 or https://nccc.ucsf.edu/clinician-consultation/pep-post-exposure-prophylaxis, or the Perinatal HIV Line at 888-448-8765 or https://nccc.ucsf.edu/clinician-consultation/perinatal-hiv-aids.

Additional information about specific exposure scenarios and resources to assist with individual determinations is available (Table 1) (Appendix A). Decision algorithms are available to provide context for the risk for HIV acquisition associated with various exposures (Table 2) (Figures 2, 3, and 4) (*16*–*19*). Expert consultation is available to assist health care professionals with assessing the HIV acquisition risk associated with different types of HIV exposure or with other questions associated with providing nPEP. Health care professionals can consult the NCCC at 888-448-4911 or https://nccc.ucsf.edu/clinician-consultation/pep-post-exposure-prophylaxis.

**TABLE 1:** Time to maximum protection estimates for daily oral pre-exposure prophylaxis with tenofovir disoproxil fumarate plus emtricitabine

Exposure	Estimated time to maximum protection for daily oral PrEP with TDF/FTC*
Receptive anal intercourse (bottoming)	7 days
Receptive vaginal intercourse	21 days^†^
Injection drug use	21 days
Insertive anal intercourse (topping)	Unknown
Insertive vaginal intercourse	Unknown

**Abbreviations:** PrEP = pre-exposure prophylaxis; TDF/FTC = tenofovir disoproxil fumarate plus emtricitabine.

* A conclusive determination of time to maximum protection for other oral PrEP or injectable PrEP medications is not yet possible from the available evidence (**Source**: Cottrell ML, Yang KH, Prince HM, et al. A translational pharmacology approach to predicting outcomes of preexposure prophylaxis against HIV in men and women using tenofovir disoproxil fumarate with or without emtricitabine. J Infect Dis 2016;214:55–64).

^†^ Preliminary pharmacokinetic data suggest earlier protection by 7 days in cervicovaginal tissues.

**TABLE 2:** Estimated per-act probability of acquiring HIV from an infected source, by exposure act

Type of exposure	Risk for HIV acquisition (per 10,000 exposures)*
**Sexual**	
Receptive anal intercourse	138
Insertive anal intercourse	11
Receptive penile-vaginal intercourse	8
Insertive penile-vaginal intercourse	4
Receptive oral intercourse	Low^§^
Insertive oral intercourse	Low^§^
**Parenteral**	
Blood transfusion	9,250
Needle sharing during injection drug use	63
Percutaneous (needle stick)	23
**Other^†^**	
Biting	Negligible
Spitting	Negligible
Sharing sex toys	Negligible

**Sources:** Patel P, Borkowf CB, Brooks JT, Lasry A, Lansky A, Mermin J. Estimating per-act HIV transmission risk: a systematic review. AIDS 2014;28:1509–19; Pretty IA, Anderson GS, Sweet DJ. Human bites and the risk of human immunodeficiency virus transmission. Am J Forensic Med Pathol 1999;20:232–9.

* Factors that might increase the risk for HIV acquisition include sexually transmitted infections, acute and late-stage HIV infection, and high viral load. Factors that might decrease the risk include condom use, male circumcision, antiretroviral treatment, and pre-exposure prophylaxis. Condomless sex is the only factor accounted for in the estimates presented.

^†^ HIV transmission through these exposure routes is technically possible but unlikely and has not been definitively demonstrated in a circumstance when contaminated body fluids (e.g., blood or sexual fluids) were absent and the exposed person’s exposure was limited to intact skin.

^§^ Risk is considered low relative to the other sexual exposures, but it is not zero; sample size in the study was too small to generate a precise point estimate.

Data are not available to determine all scenarios in which the HIV prevention benefits of nPEP exceed any potential harms. Notably, contemporary ARVs now available for nPEP regimens are both safer and more tolerable than earlier ARVs. These advances have substantially reduced potential harms associated with a time-limited exposure to these drugs when used as nPEP (*20*). Common exposure scenarios and nPEP considerations are provided (Appendix A). Each nPEP evaluation requires individual risk assessment and counseling.

#### HIV Status of the Source

If a person potentially exposed to HIV does not know the source’s HIV status, the clinician should proceed with determining whether nPEP is indicated based on the available information (Appendix A). HIV nPEP should not be delayed for the purpose of investigating the source’s HIV status. The first dose of HIV nPEP should be given to the exposed person as soon as possible. HIV nPEP should be stopped if the source is determined to not have HIV.

HIV testing for the source should be provided if they are available, are unaware or unsure of their HIV status, and agree to testing. Considerations about the type of HIV test to use for the source and testing for other STIs are similar to those considerations for evaluating persons exposed to HIV (Table 3). For persons being tested or offered testing for HIV, clinicians should also assess for behaviors associated with HIV exposure and transmission, ensure confirmatory testing is conducted, link the person to care if the test result is positive, and provide HIV PrEP counseling and services if the HIV test result is negative.

**TABLE 3:** Recommended schedule of laboratory evaluations of source and persons exposed to HIV who are evaluated for HIV nonoccupational postexposure prophylaxis

Test*	Source	Exposed			
	Baseline	Baseline	4–6 weeks after exposure	12 weeks after exposure	6 months after exposure
**All persons evaluated for nPEP**				
Rapid (point-of-care) or laboratory-based HIV Ag/Ab test^†^	X	X	X^§^	X	—
HIV diagnostic NAT^¶^	X**	X**	X^§^	X	—
HBV serology, including HBsAg, HBsAb, and HBcAb	X	X^††^	—	—	If HBV nonimmune at baseline
HCV antibody testing	—	X^§§^	—	—	If follow-up testing recommended^¶¶^
HCV RNA NAT	X***	—	If follow-up testing recommended^†††^	—	—
Syphilis serology^§§§^	X	X	X^¶¶¶^	X^¶¶¶^	—
Gonorrhea NAAT****	X****	X****	—	—	—
Chlamydia NAAT****	X****	X****	—	—	—
Pregnancy test^††††^	—	X	X	—	—
	**All persons prescribed nPEP**				
Serum creatinine		X	Only if abnormalities at baseline	—	—
Alanine transaminase and aspartate aminotransferase		X	Only if abnormalities at baseline or symptomatic	—	—

**Abbreviations:** Ag/Ab = antigen/antibody combination test; ARV = antiretroviral; HBcAb = hepatitis B core antibody; HBsAb = hepatitis B surface antibody; HBsAg = hepatitis B surface antigen; HBV= hepatitis B virus; HCV = hepatitis C virus; NAT = nucleic acid test; NAAT = nucleic acid amplification test; nPEP = nonoccupational postexposure prophylaxis; PEP = postexposure prophylaxis; STI = sexually transmitted infection.

* Any person diagnosed with an infection or condition through testing should be informed and treated or referred for treatment as needed. Recommendations are available for treatment of HIV (**Source:** Panel on Treatment of HIV During Pregnancy and Prevention of Perinatal Transmission. Recommendations for the use of antiretroviral drugs during pregnancy and interventions to reduce perinatal HIV transmission in the United States. Washington, DC: US Department of Health and Human Services; 2023. https://clinicalinfo.hiv.gov/sites/default/files/guidelines/documents/perinatal-hiv/guidelines-perinatal.pdf), HBV (**Source:** Sexually transmitted infections treatment guidelines. Atlanta, GA: US Department of Health and Human Services, CDC; 2021, https://www.cdc.gov/std/treatment-guidelines/hbv.htm and Terrault NA, Lok ASF, McMahon BJ, et al. Update on prevention, diagnosis, and treatment of chronic hepatitis B: AASLD 2018 hepatitis B guidance. Hepatology 2018;67:1560–99), HCV (**Source:** AASLD-IDSA. Recommendations for testing, managing, and treating hepatitis C. 2023. https://www.hcvguidelines.org), and STIs (**Source:** Sexually transmitted infections treatment guidelines. Atlanta, GA: US Department of Health and Human Services, CDC; 2021. https://www.cdc.gov/std/treatment-guidelines/hbv.htm).

^†^ If a rapid (point-of-care) HIV Ag/Ab test is used, a laboratory-based HIV Ag/Ab test obtained at the same time will increase diagnostic sensitivity. PEP should not be delayed awaiting laboratory results. If the preferred HIV diagnostic test is not accessible, the most sensitive available test should be used.

^§^ HIV testing 4–6 weeks post-nPEP initiation can be deferred for persons who started nPEP within 24 hours of exposure, completed the full PEP course, and are not starting PrEP at this time.

^¶^ NATs that detect HIV RNA include qualitative tests for diagnosis (e.g., HIV-1 RNA assay) and quantitative tests for disease monitoring (e.g., viral load). Diagnostic HIV NATs are recommended because they are more likely than viral load tests to detect very low levels of HIV. If the preferred HIV diagnostic test is not accessible, the most sensitive available test should be used; inability to access HIV NAT should not prevent provision of HIV nPEP to persons with indications.

** HIV NAT recommended at baseline assessment for persons with injectable ARV exposure during the past 6 months.

^††^ HBV PEP recommendations vary by the exposed person’s HBV immune status, and by the source’s HBV status (when information available). Recommendations are available for HBV postexposure management (**Source:** Schillie S, Vellozzi C, Reingold A, et al. Prevention of hepatitis B virus infection in the United States: recommendations of the Advisory Committee on Immunization Practices. MMWR Recomm Rep 2018;67:1–31).

^§§^ Reflex to HCV RNA NAT if HCV antibody test is positive. Add HCV RNA NAT to original order if signs and symptoms of acute HCV infection are present (e.g., hepatic enzyme elevation).

^¶¶^ If follow-up testing is recommended based on the source’s status (e.g., HCV RNA positive or HCV antibody test is positive with unavailable HCV RNA, or if the HCV infection status is unknown), and HCV RNA NAT is negative 3–6 weeks postexposure, a final test for HCV antibodies 4–6 months postexposure is recommended (**Source:** Moorman AC, de Perio MA, Goldschmidt R, et al. Testing and clinical management of health care personnel potentially exposed to hepatitis C virus—CDC guidance, United States, 2020. MMWR Recomm Rep 2020;69:1–8).

*** HCV RNA NAT is preferred for testing of the source, but if not accessible, HCV antibody testing with reflex HCV RNA NAT if positive is an alternative strategy (**Source:** Moorman AC, de Perio MA, Goldschmidt R, et al. Testing and clinical management of health care personnel potentially exposed to hepatitis C virus—CDC guidance, United States, 2020. MMWR Recomm Rep 2020;69:1–8).

^†††^ If follow-up testing is recommended based on the source’s status (e.g., HCV RNA positive or positive HCV antibody with unavailable HCV RNA, or if the HCV infection status is unknown), HCV RNA NAT is recommended for the exposed persons 3–6 weeks postexposure (**Source:** Moorman AC, de Perio MA, Goldschmidt R, et al. Testing and clinical management of health care personnel potentially exposed to hepatitis C virus—CDC guidance, United States, 2020. MMWR Recomm Rep 2020;69:1–8).

^§§§^ STI testing decisions should be made on an individual basis. Recommendations are available for presumptive STI treatment, testing, and management after assault or possible acute STI exposure (**Source:** CDC. Sexually transmitted infections treatment guidelines. Atlanta, GA: US Department of Health and Human Services, CDC; 2021. https://www.cdc.gov/std/treatment-guidelines/default.htm).

^¶¶¶^ If initial syphilis testing negative and infection in the source cannot be ruled out, follow-up testing may be performed 4–6 weeks and 3 months postexposure.

**** NAATs are recommended for *Chlamydia trachomatis* and *Neisseria gonorrhoeae* at exposure sites (e.g., pharynx, rectum, or vagina) at initial visit and can be repeated 1–2 weeks postexposure if no presumptive treatment was provided and initial test results were negative. Repeat testing can also be done if the person reports symptoms concerning for STIs. Certain experts would also perform a NAAT for *Trichomonas vaginalis* from a urine or vaginal specimen for persons with vaginas (**Source:** CDC. Sexual assault and abuse and STIs — adolescents and adults 2021. Atlanta, GA: US Department of Health and Human Services, CDC; 2021. https://www.cdc.gov/std/treatment-guidelines/sexual-assault-adults.htm).

^††††^ For all women of child-bearing potential who are not known to be pregnant.

If the source is known to have HIV, information about their current viral load and adherence with antiretroviral therapy (ART), history of ARV treatment regimens, and HIV resistance testing are relevant for the care of the exposed person. If the source consents to share this information and it is immediately available at the time of nPEP evaluation, the clinician should consider whether the source has sustained viral suppression and, therefore, is not expected to transmit HIV (Appendix A). When nPEP is indicated, clinicians should consider all available information as described to design an nPEP regimen most likely to be active against the HIV to which the person was exposed (see HIV nPEP Regimens).

#### nPEP in the Context of PrEP and PrEP Adherence

A pharmacokinetics study among gay, bisexual, and other men who have sex with men (GBMSM) taking tenofovir disoproxil fumarate (TDF) as PrEP estimated that HIV risk reduction efficacy was 99% for ≥4 doses per week and 84% for 2–3 doses per week (*21*–*23*). A laboratory study measuring vaginal tissue levels of active metabolites of TDF and emtricitabine (FTC) found that drug levels associated with significant protection against HIV infection required 6–7 doses per week (>85% adherence) for lower vaginal tract tissues (*24*). When data are limited (e.g., for injectable PrEP and intermittent PrEP), it might be prudent to consider any pattern of use outside current guideline recommendations as being nonadherent and offer persons with HIV exposure PEP while nonadherent on PrEP (*5*). Persons with repeat or continuing exposure to HIV after the end of the nPEP course should be offered PrEP to reduce their risk for acquiring HIV.

### Time to Initiation of HIV nPEP

Exposure to HIV is a medical emergency. When indicated, nPEP should be initiated as soon as possible during the clinical encounter with confirmed linkage to follow-up care Available clinical evidence suggests that the shorter the time from HIV exposure to nPEP initiation, the greater the likelihood of preventing HIV acquisition (*25*).

#### Recommendation for Time to Initiation of HIV nPEP

- Initiate nPEP as soon as possible, but no later than 72 hours after exposure (**good practice statement, existing recommendation**).

#### Rationale for Time to Initiation of HIV nPEP

The 72-hour initiation window for nPEP was established through non-human primate studies that demonstrated decreasing PEP efficacy with increasing time between exposure and ARV initiation. One study of macaques with vaginal exposure to HIV-2 demonstrated that all animals given non-oral PEP (subcutaneous TDF for 28 days) started 12 or 36 hours postexposure remained uninfected, in contrast to three of four untreated control animals who acquired HIV-2 infection (*26*). In the group of animals administered PEP 72 hours postexposure, one animal died of causes unrelated to the study. Among the three surviving animals, one acquired HIV-2 infection. A study of rhesus monkeys with rectal exposure to simian immunodeficiency virus demonstrated that on withdrawal of ARVs 6 months postexposure, viral rebound occurred in 0% (zero of five), 20% (one of five), 60% (three of five), and 100% (five of five) of animals that initiated ARVs on day 0 (6 h), 1, 2, or 3, respectively (*27*).

Evidence is insufficient to recommend nPEP initiation later than 72 hours postexposure. However, certain experts have argued that risk versus benefit considerations could favor a longer initiation window. Newer ARV regimens could possibly prevent establishment of HIV infection >72 hours after exposure, and current preferred nPEP regimens are generally safe and well tolerated (*20*). CDC’s HIV oPEP guidelines provide discussion for health care professionals who might be considering PEP initiation >72 hours after HIV exposure (*5*). Persons seeking nPEP care >72 hours after an HIV exposure should be tested for HIV, provided HIV prevention counseling including PrEP education, and provided with a tailored follow-up plan including follow-up HIV testing (*2*,*28*).

### HIV nPEP Regimens

A 28-day course of nPEP is recommended for persons without HIV who seek care ≤72 hours after a nonoccupational exposure to blood, genital secretions, or other potentially infectious body fluids of persons known to have HIV or of unknown HIV status when that exposure represents a substantial risk for HIV acquisition (*25*–*27*,*29*–*32*). The goal of nPEP is to provide a potent, safe, tolerable, and easy-to-adhere-to regimen to reduce the risk for HIV transmission and viral replication. The choice of regimen should be guided by the regimen’s potency and efficacy, barrier to resistance, adverse effects profile, convenience, the patient’s comorbidities and concomitant medications, and the potential for drug–drug interactions. Because adherence is critical for nPEP efficacy, selection of regimens that minimize side effects, the number of doses per day, and the number of pills per dose is preferable.

 A quick reference for dosing and basic information for ARV medications in both preferred and alternative nPEP regimens is available (Table 4), as is guidance about preferred and alternative regimens for adults and adolescents and regimens for children, pregnant women, and persons with renal or hepatic dysfunction (Tables 5 and 6). Selection of a regimen should be individualized based on comorbid conditions (e.g., renal or hepatic dysfunction), pregnancy, drug interaction potential with concurrent medications, previous exposure to ARV regimens (including long-acting injectable ARVs), the source’s history, and regimen factors that might influence adherence (e.g., pill burden, dosing frequency, side effects, cost, and access). Prescribing information including dosing and potential drug interactions can be found in the manufacturers’ package inserts, the National Institute of Health’s (NIH’s) Guidelines for the Use of Antiretroviral Agents in Adults and Adolescents (https://clinicalinfo.hiv.gov/en/guidelines/hiv-clinical-guidelines-adult-and-adolescent-arv/whats-new), and the University of Liverpool HIV drug interaction checker (https://www.hiv-druginteractions.org).

**TABLE 4:** Preferred and alternative HIV nonoccupational postexposure prophylaxis regimens, by group — CDC recommendations, United States, 2025

Group	Preferred/Alternative	Regimen*^,†,§^
**Adults and adolescents aged ≥12 yrs**	**Preferred**	**Integrase strand transfer inhibitors PLUS two nucleoside reverse transcriptase inhibitors** • Bictegravir/emtricitabine/tenofovir alafenamide, OR • Dolutegravir PLUS (tenofovir alafenamide OR tenofovir disoproxil fumarate) PLUS (emtricitabine OR lamivudine)
	Alternative	**Boosted protease inhibitor PLUS two nucleoside reverse transcriptase inhibitors** • Darunavir and cobicistat OR darunavir and ritonavir PLUS (tenofovir alafenamide OR tenofovir disoproxil fumarate) PLUS (emtricitabine OR lamivudine)
**Pregnant women**	**Preferred**	**Integrase strand transfer inhibitors PLUS two nucleoside reverse transcriptase inhibitors** • Bictegravir/emtricitabine/tenofovir alafenamide, OR • Dolutegravir PLUS (tenofovir alafenamide OR tenofovir disoproxil fumarate) PLUS (emtricitabine OR lamivudine)
	Alternative	**Integrase strand transfer inhibitors PLUS two nucleoside reverse transcriptase inhibitors** **Boosted protease inhibitor PLUS two nucleoside reverse transcriptase inhibitors** • Darunavir and ritonavir (twice daily) PLUS (tenofovir alafenamide OR tenofovir disoproxil fumarate) PLUS (emtricitabine OR lamivudine)
**Children aged ≥2 yrs to 12 yrs**	**Preferred**	**Integrase strand transfer inhibitors PLUS two nucleoside reverse transcriptase inhibitors** • Bictegravir/emtricitabine/tenofovir alafenamide (≥14 kg),^¶ ^OR • Dolutegravir PLUS (tenofovir alafenamide OR tenofovir disoproxil fumarate) PLUS (emtricitabine OR lamivudine)
	Alternative	**Boosted protease inhibitor PLUS two nucleoside reverse transcriptase inhibitors** • Darunavir and ritonavir (aged ≥3 yrs and ≥10 kg) PLUS (tenofovir alafenamide OR tenofovir disoproxil fumarate) PLUS (emtricitabine OR lamivudine),** OR • Lopinavir and ritonavir PLUS (tenofovir alafenamide OR tenofovir disoproxil fumarate) PLUS (emtricitabine OR lamivudine)^¶^
**Infants and children aged ≥4 weeks to 2 yrs**	**Preferred**	**Integrase strand transfer inhibitors PLUS two nucleoside reverse transcriptase inhibitors** • Dolutegravir (>3 kg) PLUS zidovudine PLUS (emtricitabine OR lamivudine)
	Alternative	**Integrase strand transfer inhibitors PLUS two nucleoside reverse transcriptase inhibitors** • Raltegravir (≥2 kg) PLUS zidovudine PLUS (emtricitabine OR lamivudine) **Boosted protease inhibitor PLUS two nucleoside reverse transcriptase inhibitors** • Lopinavir and ritonavir PLUS zidovudine PLUS (emtricitabine OR lamivudine)
**Neonates aged ≥14 days to <4 weeks**	Not applicable	Consult perinatal prevention guidelines for ARV recommendations for newborns with known or possible perinatal HIV exposure (https://clinicalinfo.hiv.gov/en/guidelines/perinatal/whats-new). For all other scenarios consult a pediatric HIV specialist using local resources or the NCCC PEPline at 888-448-4911 or https://nccc.ucsf.edu/clinician-consultation/pep-post-exposure-prophylaxis.

**Abbreviations**: ARV = antiretroviral; FDC = fixed-dose combination; NCCC = National Clinician Consultation Center; nPEP = nonoccupational postexposure prophylaxis.

* The following are available as single tablet complete nPEP regimens: bictegravir/emtricitabine/tenofovir alafenamide (BIC/FTC/TAF; a preferred regimen) and darunavir/cobicistat/tenofovir alafenamide /emtricitabine (DRV/c/TAF/FTC; an alternative regimen). Generic drug forms are available for darunavir (DRV), ritonavir (RTV), tenofovir disoproxil fumarate (TDF)/emtricitabine (FTC), TDF/lamivudine (3TC), and 3TC. See Table 6 for additional prescribing information including other combination products. Antiviral potency, tolerability, client preferences, cost, and access are all considerations when selecting an nPEP regimen. Health care professionals unfamiliar with these medications should use local infectious diseases or other expert consultation resources or consult the NCCC PEPline at 888-448-4911 or https://nccc.ucsf.edu/clinician-consultation/pep-post-exposure-prophylaxis or the Perinatal HIV Line at 888-448-8765 or https://nccc.ucsf.edu/clinician-consultation/perinatal-hiv-aids.

^†^ Regimens within categories are listed in alphabetical order and not according to preference.

^§^ All listed medications in this table are off-label use for nPEP.

^¶^ Bictegravir (BIC) is available only as part of a FDC tablet that contains BIC/TAF/FTC; this FDC tablet is recommended as a preferred regimen for children aged ≥2 years and weighing ≥14 kg. Two strengths of BIC/TAF/FTC are available, with dosing according to a child’s weight.

** TAF/FTC should not be used with a boosted protease inhibitor if weight is <35kg.

**TABLE 5:** Preferred and alternative HIV nonoccupational postexposure prophylaxis regimens for patients with renal dysfunction or hepatic impairment, for use with expert consultation — CDC recommendations, United States, 2025

Group	Preferred/Alternative	Regimen*^,†,§^
**Adults and adolescents aged ≥12 yrs, with moderate renal dysfunction (CrCl 30–49 mL/min)**	**Preferred**	**Integrase strand transfer inhibitors PLUS two nucleoside reverse transcriptase inhibitors** • Bictegravir/tenofovir alafenamide/emtricitabine, OR • Dolutegravir PLUS tenofovir alafenamide PLUS (emtricitabine OR lamivudine^¶^)
	Alternative	**Integrase strand transfer inhibitors PLUS two nucleoside reverse transcriptase inhibitors** • Dolutegravir PLUS dose-reduced tenofovir disoproxil fumarate**^,††^ PLUS (emtricitabine OR lamivudine^¶^) **Boosted protease inhibitor PLUS two nucleoside reverse transcriptase inhibitors** • Darunavir/cobicistat/tenofovir alafenamide/emtricitabine, OR • Darunavir and ritonavir PLUS (tenofovir alafenamide OR dose-reduced tenofovir disoproxil fumarate**^,††^) PLUS (emtricitabine OR lamivudine^††^)
**Adults and adolescents aged ≥12 yrs, with severe renal dysfunction (CrCl <30 mL/min) and on hemodialysis**	**Preferred**	**Integrase strand transfer inhibitors PLUS two nucleoside reverse transcriptase inhibitors** • Bictegravir/tenofovir alafenamide/emtricitabine, OR • Dolutegravir PLUS tenofovir alafenamide PLUS (emtricitabine OR dose-reduced lamivudine^††^)
	Alternative	**Integrase strand transfer inhibitors PLUS two nucleoside reverse transcriptase inhibitors** • Dolutegravir PLUS dose-reduced tenofovir disoproxil fumarate** PLUS (emtricitabine OR dose-reduced lamivudine^††^) **Boosted protease inhibitor PLUS two nucleoside reverse transcriptase inhibitors** • Darunavir/cobicistat/tenofovir alafenamide/emtricitabine, OR • Darunavir and ritonavir PLUS (tenofovir alafenamide OR dose-reduced tenofovir disoproxil fumarate**^,††^) PLUS (emtricitabine OR dose-reduced lamivudine^††^)
**Adults and adolescents aged ≥12 yrs, with severe renal dysfunction (CrCl <30 mL/min), not on hemodialysis**	Not applicable	Consult HIV specialist Health care professionals should consult a local HIV specialist or consult the NCCC PEPline at 888-448-4911 or https://nccc.ucsf.edu/clinician-consultation/pep-post-exposure-prophylaxis
**Adults and adolescents aged ≥12 yrs, with hepatic impairment (Child-Pugh class A or B)**	**Preferred**	**Integrase strand transfer inhibitors PLUS two nucleoside reverse transcriptase inhibitors**^†^ • Bictegravir/tenofovir alafenamide/emtricitabine, OR • Dolutegravir PLUS (tenofovir alafenamide OR tenofovir disoproxil fumarate) PLUS (emtricitabine OR lamivudine)
	Alternative	**Boosted protease inhibitor PLUS two nucleoside reverse transcriptase inhibitors** • Darunavir and cobicistat OR darunavir and ritonavir PLUS (tenofovir alafenamide OR tenofovir disoproxil fumarate) PLUS (emtricitabine OR lamivudine)
**Adults and adolescents aged ≥12 yrs, with hepatic impairment (Child-Pugh class C)**	Not applicable	Consult HIV specialist

**Abbreviations:** CrCl = creatinine clearance; FDC = fixed-dose combination; NCCC = National Clinician Consultation Center; nPEP = nonoccupational postexposure prophylaxis.

* Regimens within categories are listed in alphabetical order and not according to preference.

^†^ The following are available as single tablet complete nPEP regimens: bictegravir/tenofovir alafenamide/emtricitabine (BIC/TAF/FTC; a preferred regimen) and darunavir/cobicistat/tenofovir alafenamide /emtricitabine (DRV/c/TAF/FTC; an alternative regimen). Generic drug forms are available for darunavir (DRV), ritonavir (RTV), tenofovir disoproxil fumarate (TDF)/emtricitabine (FTC), TDF/lamivudine (3TC), and 3TC. See Table 6 for additional prescribing information including other combination products. Antiviral potency, tolerability, client preferences, cost, and access are all considerations when selecting an nPEP regimen. Certain single tablet regimens might be inappropriate for persons with organ dysfunction. Health care professionals unfamiliar with these medications should use local infectious diseases or other expert consultation resources or consult the NCCC PEPline at 888-448-4911 or https://nccc.ucsf.edu/clinician-consultation/pep-post-exposure-prophylaxis or the Perinatal HIV Line at 888-448-8765 or https://nccc.ucsf.edu/clinician-consultation/perinatal-hiv-aids.

^§^ All listed medications in this table are off-label use for nPEP.

^¶^ The prescribing information for lamivudine recommends dosage adjustment from 300 mg once daily to 150 mg once daily for patients with CrCl 30–49 mL/min. However, the prescribing information for multiple FDC products that contain lamivudine recommends no dose adjustment for CrCl 30–49 mL/min. Therefore, no dose adjustment is needed for lamivudine when administered as a standalone tablet or part of an FDC tablet.

** TDF 300 mg every 48 hours.

^††^ Please see manufacturer’s package insert for dosing instructions for individual agents or consult the antiretroviral dosing recommendations in adults with renal or hepatic insufficiency (https://clinicalinfo.hiv.gov/en/guidelines/hiv-clinical-guidelines-adult-and-adolescent-arv/drug-characteristics-tables-renal-hepatic-insufficiency-full).

**TABLE 6:** Formulations, cautions, and dosing considerations for antiretroviral medications in preferred and alternative nonoccupational postexposure prophylaxis regimens — CDC recommendations, United States, 2025

Drug and available formulation*^,†^	Standard adult dosing	Drug administration, most common side effects, interactions, contraindications, and cautions	Dosing adjustments
**Combination product**			
Bictegravir/emtricitabine/tenofovir alafenamide (Biktarvy, Gilead Sciences, Inc., BIC/FTC/TAF) **Formulation:** BIC 50 mg/FTC 200mg/TAF 25 mg tablet BIC 30 mg/FTC 120 mg/TAF 15 mg tablet	BIC 50 mg/FTC 200 mg/TAF 25 mg once daily by mouth	**Administration:** Take with or without food; administer BIC/FTC/TAF 2 hours before or 6 hours after medications containing polyvalent cations, such as aluminum, calcium, iron, and magnesium iron (alternatively, calcium and iron can be taken with BIC/FTC/TAF with food); tablet can be split with each part taken separately; both parts should be ingested within 10 minutes **Most common side effects:** Diarrhea, nausea, headache **Drug interactions:** Screen for drug interactions^†^ **Contraindications:** Do not take with dofetilide or rifampin **Cautions:** Potential exacerbation of HBV infection upon initiation or discontinuation of treatment; bictegravir increases serum creatinine without affecting glomerular filtration rate	**Renal:** eCrCl ≥30 mL/min: • Use standard dose eCrCl <30 mL/min and not on HD: • Not recommended On HD: • Use standard dose, administer after HD on dialysis days **Hepatic:** Child-Pugh class A or B: • Use standard dose Child-Pugh class C: • Not recommended **Pregnancy:** Use standard dose **Pediatric:** Weight >25 g: • Use standard dose Weight ≥14–25 kg: • BIC 30 mg/FTC 120 mg/TAF 15 mg once daily by mouth Weight <14 kg: • Not recommended
Darunavir/cobicistat/emtricitabine/tenofovir alafenamide (Symtuza, Janssen, DRV/COBI/FTC/TAF) **Formulation:** DRV 800 mg/COBI 150 mg/FTC 200 mg/TAF 10 mg tablet	DRV 800 mg/COBI 150 mg/FTC 200 mg/TAF 10 mg by mouth once daily	**Administration:** Take with food; tablet may be split in half, but both parts should be consumed immediately after splitting **Most common side effects:** Diarrhea, rash, nausea, fatigue, headache, abdominal discomfort, flatulence **Drug interactions:** Screen for drug interactions^†^ **Contraindications:** Inhibits CYP3A enzyme resulting in many contraindications with medications; drug interaction review is critical **Cautions:** Risk for Stevens-Johnson syndrome and toxic epidermal necrosis; use with caution in persons with a sulfonamide allergy; may cause drug-induced hepatitis; potential exacerbation of HBV infection upon initiation or discontinuation of HBV treatment; cobicistat increases serum creatinine without affecting glomerular filtration rate	**Renal:** eCrCl ≥30 mL/min: • Use standard dose eCrCl <0 mL/min and not on HD: • Not recommended On HD: • Use standard dose; administer after HD on dialysis days **Hepatic:** Child-Pugh class A and B: • Use standard dose Child-Pugh class C: • Not recommended **Pregnancy:** Not recommended **Pediatric:** Weight ≥40 kg: • Use standard dose Weight <40 kg: • Not recommended
Darunavir/cobicistat (Prezcobix, Janssen, DRV/COBI) **Formulation:** DRV 800 mg/COBI 150 mg tablet	DRV 800 mg/COBI 150 mg once daily by mouth	**Administration:** Take with food **Most common side effects:** Diarrhea, nausea, headache **Drug interactions:** Screen for drug interactions^†^ **Contraindications:** Inhibits CYP3A enzyme resulting in many contraindications with medications; drug interaction review is critical **Cautions:** Risk for Stevens-Johnson syndrome and toxic epidermal necrosis; can cause hepatotoxicity; use with caution in persons with a sulfonamide allergy; cobicistat increases serum creatinine without affecting glomerular filtration rate	**Renal:** • Use standard dose • Do not co-administer with TDF for eCrCl <70 mL/min **Hepatic:** Child-Pugh class A or B: • Use standard dose Child-Pugh class C: • Not recommended **Pregnancy:** Not recommended (see DRV/r) **Pediatric:** Weight ≥40 kg: • Use standard dose Weight <40kg: • Not recommended (see DRV/r)
Emtricitabine/tenofovir alafenamide (Descovy, Gilead Sciences, Inc., FTC/TAF) **Formulation:** FTC 200 mg/TAF 25 mg tablet FTC 120 mg/TAF 15 mg tablet	FTC 200 mg/TAF 25 mg	**Administration:** Take with or without food **Most common side effects:** Nausea, diarrhea, abdominal pain, fatigue, headache **Drug interactions:** Screen for drug interactions^†^ **Contraindications:** None **Cautions:** Potential exacerbation of HBV infection upon initiation or discontinuation of treatment	**Renal:** eCrCl ≥30 mL/min: • Use standard dose eCrCl <30 mL/min and not on HD: • Not recommended On HD: • Use standard dose; administer after HD on dialysis days **Hepatic:** Child-Pugh class A or B: • Use standard dose Child-Pugh class C: • Not recommended **Pregnancy:** Use standard dose **Pediatric:** Weight ≥25kg: • Use standard dose Weight 14–25 kg: • FTC 120 mg/15 mg TAF once daily Weight <14 kg: • Not recommended (FTC/TAF should not be used with a boosted protease inhibitor [darunavir with ritonavir or cobicistat OR Lopinavir with ritonavir] for patients weighing <35kg)
Emtricitabine/tenofovir disoproxil fumarate (Truvada, Gilead Sciences, Inc., FTC/TDF) **Truvada formulation:** FTC 200 mg/TDF 300 mg tablet FTC 167 mg/TDF 250 mg tablet FTC 133 mg/TDF 200 mg tablet FTC 100 mg/TDF 150 mg tablet **Generic formulation:** FTC 200 mg/TDF 300 mg tablet FTC 167 mg/TDF 250 mg tablet FTC 133 mg/TDF 200 mg tablet FTC 100 mg/TDF 150 mg tablet	FTC 200 mg/TDF 300 mg daily by mouth	**Administration:** Take with or without food **Most common side effects:** Nausea, diarrhea, headache, fatigue **Drug interactions:** screen for drug interactions^†^ **Contraindications:** None **Cautions:** Potential exacerbation of HBV infection upon initiation or discontinuation of treatment, new onset renal impairment	**Renal:** eCrCl ≥50 mL/min: • Use standard dose eCrCl 30–49 mL/min: • FTC 200 mg/TDF 300 mg every 48 hours eCrCl <30 mL/min or on HD: • Not recommended (see single-drug products) **Hepatic:** Use standard dose **Pregnancy:** Use standard dose **Pediatric:** Weight >35 kg: • Use standard dose Weight 28 kg to <35kg: • One tablet FTC 167 mg/TDF 250 mg daily Weight 22 kg to <28kg: • One tablet FTC 133 mg/TDF 200 mg daily Weight 17 kg to < 22 kg: • One tablet FTC 100 mg/TDF 150 mg daily Weight <17 kg: • Not recommended
Lopinavir/ritonavir (Kaletra, AbbVie Inc., LPV/r) **Formulation:** LPV 200/r 50 mg tablet LPV 100/r 25 mg tablet LPV 80/r 20 mg/mL oral solution	Not recommended for nPEP in adults (see Tables 5 and 6 for recommendations)	**Administration:** • Tablet: Take with or without food; do not chew, break, or crush tablets • Oral solution (42.4% alcohol): Take with food **Most common side effects:** Nausea, vomiting, diarrhea, dysgeusia **Drug interactions:** Screen for drug interactions^†^ **Contraindications:** Inhibits CYP3A enzyme resulting in many contraindications with medications, drug interaction review is critical **Cautions:** PR and QT interval prolongation have been reported; use with caution with patients at risk for cardiac conduction abnormalities or receiving other drugs with similar effect; health care professionals should be aware that the oral solution is highly concentrated and contains 42.4% alcohol (by volume) and 15.3% propylene glycol (by weight/volume)	**Pediatric:** Aged 6 mos–18 yrs, weight-based dosing, LPV/r tablet: • Weight >35 kg: Four LPV 100/r 25 mg tablets twice daily or two LPV 200/r 50 mg tablets twice daily • Weight >25 to 35 kg: Three LPV 100/r 25 mg tablets twice daily • Weight 15–25 kg: Two LPV 100/r 25 mg tablets twice daily • Weight <15 kg: Not recommended Aged 6 mos–18 yrs, weight-based dosing (not to exceed the recommended adult dose), LPV/r oral solution: • Weight >40 kg: LPV 400/r 100 mg twice daily • Weight ≥15 kg to 40 kg: LPV 10/r 2.5 mg/kg twice daily • Weight <15 kg: LPV 12/r 3 mg/kg twice daily Aged 14 days to <6 mos (not recommended for neonates before a postmenstrual age [first day of the mother’s last menstrual period to birth plus the time elapsed after birth] of ≥42 weeks), weight-based or body surface area dosing, LPV/r oral solution: • LPV 16/r 4 mg/kg or LPV 300/r 75 mg/m^2^ twice daily Aged <14 days • Not recommended
Tenofovir disoproxil fumarate/lamivudine (Cimduo, Mylan Pharmaceuticals Inc. (generic), TDF/3TC) **Formulation:** TDF 300 mg/3TC 300 mg tablet	TDF 300 mg/3TC 300 mg daily by mouth	**Administration:** Take with or without food **Most common side effects:** Diarrhea, nausea, headache, fatigue, malaise, neuropathy, insomnia, rash **Drug interactions:** Screen for drug interactions^†^ **Contraindications:** None **Cautions:** Potential exacerbation of HBV infection upon discontinuation or initiation of therapy, new onset renal impairment	**Renal:** eCrCl ≥50 mL/minute: • Use standard dose eCrCl <50 mL/minute or on HD: • Not recommended (see single-drug product) **Hepatic:** Use standard dose **Pregnancy:** Use standard dose **Pediatric:** Weight ≥35 kg: • Use standard dose Weight <35 kg: • Not recommended (see single drug products)
**Single-drug product**			
Darunavir (Prezista, Janssen Therapeutics, DRV) Always use with ritonavir (r or RTV) or cobicistat (COBI) **Formulation:** 800 mg tablet 600 mg tablet 150 mg tablet 75 mg tablet 100 mg/mL oral suspension	DRV 800 mg with RTV 100 mg once daily by mouth	**Administration:** • Tablet: Take with food • Suspension: Shake well before use; take with food **Most common side effects:** Diarrhea, nausea, headache **Drug interactions:** Screen for drug interactions^†^ **Contraindications:** Inhibits CYP3A enzyme resulting in many contraindications with medications, drug interaction review is critical **Cautions:** DRV must be co-administered with RTV or COBI; risk for Stevens-Johnson syndrome and toxic epidermal necrosis; can cause hepatotoxicity; use with caution in persons with known allergy to sulfonamide medications	**Renal:** Use standard dose **Hepatic:** Child-Pugh class A and B: • Use standard dose Child-Pugh class C: • Not recommended **Pregnancy:** DRV 600 mg with RTV 100 mg twice daily **Pediatric:** Aged 3 to <18 yrs, weight-based dosing: • Weight ≥40 kg: Use standard dose • Weight 30 to <40 kg: DRV 675 mg/6.8 mL with RTV 100 mg/1.25 mL (RTV 80 mg/mL oral solution) once daily by mouth • Weight 15 to <30 kg: DRV 600 mg/6 mL with RTV 100 mg/1.25 mL (RTV 80 mg/mL oral solution) once daily by mouth • Weight 10 kg to <15 kg: DRV 35 mg/kg with RTV 7 mg/kg once daily by mouth Aged <3 yrs OR weight <10 kg: • Not recommended
Dolutegravir (Tivicay, ViiV Healthcare, DTG) **Tivicay formulation:** 50 mg tablet 25 mg tablet 10 mg tablet **Tivicay PD tablet for oral suspension formulation:** 5 mg tablet	50 mg tablet once daily by mouth	**Administration:** • All formulations: Take with or without food; administer DTG 2 hours before or 6 hours after medications containing polyvalent cations such as magnesium, aluminum, calcium, and iron (alternatively, calcium and iron can be taken with DTG and food) • Tablets must be swallowed whole; do not chew, crush, or cut Tivicay PD; fully disperse up to 3 tablets in 5 mL of water and swirl until no lumps remain, administer oral suspension within 30 minutes **Most common side effects:** Insomnia, fatigue, headache **Drug interactions:** Screen for drug interactions^†^ **Contraindications:** Do not administer with dofetilide **Cautions:** Most drug cautions unlikely to occur in the setting of nPEP; DTG increases serum creatinine without affecting glomerular filtration rate	**Renal:** Use standard dose **Hepatic:** Child-Pugh class A or B: • Use standard dose Child-Pugh class C: • Not recommended **Pregnancy:** Use standard dose **Pediatric:** Aged <12 yrs, weight-based dosing, Tivicay tablet: • Weight >20 kg: 50 mg daily by mouth • Weight 14 kg to <20 kg: 40 mg daily by mouth • Weight <14 kg: Not recommended (see Tivicay PD for oral suspension) Aged ≥4 weeks to 11 yrs, weight-based dosing, using Tivicay PD for oral suspension: • Weight 20 kg to <40kg: 30 mg daily by mouth • Weight 14 kg to <20 kg: 25 mg daily by mouth • Weight 10 kg to <14 kg: 20 mg daily by mouth • Weight 6 kg to <10 kg: 15 mg daily by mouth • Weight 3 kg to <6kg: 5 mg daily by mouth Aged <4 weeks OR weight <3 kg: • Not recommended
Emtricitabine (Emtriva, Gilead Sciences, Inc., FTC) **Formulation:** 200 mg capsule 10 mg/mL oral solution Dose of capsule and oral suspension is not interchangeable	Capsule: 200 mg once daily by mouth OR Oral solution: 240 mg/24 mL once daily by mouth	**Administration:** Take with or without food **Most common side effects:** Hyperpigmented rash or skin discoloration **Drug interactions:** Screen for drug interactions^†^ **Contraindications:** None **Cautions:** Potential exacerbation of HBV infection upon discontinuation or initiation of treatment	**Renal:** eCrCl ≥30 mL/min • Use standard dose eCrCl 15–29 mL/min: • Capsule: 200 mg every 72 hours • Solution: 80 mg/8 mL every 24 hours eCrCl <15 mL/minute and not on HD: • Capsule: 200 mg every 96 hours • Solution: 60 mg/6 mL every 24 hours On HD: • Use standard dose, dose after HD on dialysis days **Hepatic:** Use standard dose **Pregnancy:** Use standard dose **Pediatric:** Weight-based dosing, FTC capsule: • Weight ≥33 kg: 200 mg tablet once daily by mouth • Weight <33 kg: Not recommended Aged 3 mos–17 yrs, weight-based dosing, FTC (oral solution): • 6 mg/kg once daily (oral solution) by mouth (not to exceed 240 mg oral solution once daily) Aged 0–3 mos, weight-based dosing, FTC (oral solution): • 3 mg/kg once daily by mouth
Lamivudine (Epivir, ViiV Healthcare, 3TC) **Formulation:** 150 mg scored tablet 300 mg tablet 100 mg tablet 10 mg/mL oral solution	300 mg once daily by mouth OR 150 mg twice daily by mouth	**Administration:** Take with or without food **Most common side effects:** Headache, nausea, malaise, fatigue **Drug interactions:** Screen for drug interactions^†^ **Contraindications:** None **Cautions:** Potential exacerbation of HBV infection upon initiation or discontinuation of treatment	**Renal:** eCrCl ≥30 mL/min • Use standard dose eCrCl 15–29 mL/min and not on HD: • 150 mg once then 100 mg daily eCrCl 5–14 mL/min and not on HD: • 150 mg once then 50 mg daily eCrCl <5 mL/min and not on HD: • 50 mg once then 25 mg daily On HD: • 50 mg once then 25 mg daily, administer after HD on dialysis days **Hepatic:** Use standard dose **Pregnancy:** Use standard dose **Pediatric:** Aged ≥16 yrs, weight-based dosing: • Weight ≥50 kg: 150 mg twice daily or 300 mg once daily • Weight <50 kg: 4 mg/kg (up to 150 mg) twice daily Aged <16 yrs and weight ≥14 kg, scored 150 mg tablet: • Weight ≥25 kg: 150 mg tablet twice daily • Weight 20 to <25 kg: 75 mg (1/2 tablet) a.m. PLUS 150 mg (1 tablet) p.m. • Weight 14 to <20 kg: 75 mg (1/2 tablet) a.m. PLUS 75 mg (1/2 tablet) p.m. • Weight <14 kg: Not recommended Aged >3 mos, weight-based dosing, oral solution: • 5 mg/kg twice a day OR • 10 mg/kg once daily (maximum dose of 300 mg daily) Aged ≥4 weeks to <3 mos, weight-based dosing, oral solution: • 4 mg/kg (maximum dose 150 mg) twice daily Aged ≤27 days to <4 weeks, weight-based dosing, oral solution: • 2 mg/kg twice daily
Raltegravir (Isentress, Merck & Co., Inc., RAL) **Isentress formulation:** 400 mg tablet 100 mg chewable, scored tablet 25 mg chewable tablet 100 mg single-use packet for oral suspension Dose of tablet with chewable tablet and oral suspension is not interchangeable **Isentress HD formulation:** 600 mg tablet	Not recommended for nPEP in adults (see Tables 5 and 6 for recommendations)	**Administration:** • All formulations: Take with or without food; administer RAL 2 hours before or 6 hours after medications containing polyvalent cations such as magnesium, aluminum, calcium, and iron • Isentress HD tablet: Swallow whole • Chewable tablet: Chew, swallow whole, crush, or dissolve using 5 mL of water, juice, or breastmilk; take dose within 2 minutes • Oral suspension: Do not open packet until ready to use; dissolve packet in 10 mL of water, swirl for 45 seconds to create 10 mg/mL solution; administer dose within 30 minutes **Most common side effects:** Insomnia, nausea, fatigue, headache **Drug interactions**: Screen for drug interactions^†^ **Contraindications:** None **Cautions: Risk for** Stevens-Johnson syndrome and toxic epidermal necrosis	**Pediatric:** Aged 6–12 yrs and weight >25 kg, tablet: • 400 mg tablet twice daily Aged 2–12 yrs, weight-based dosing, chewable tablet: • Weight >40 kg: 300 mg twice daily • Weight 28 to <40 kg: 200 mg twice daily • Weight 20 to <28 kg: 150 mg twice daily • Weight 14 to <20 kg: 100 mg twice daily • Weight 11 to <14 kg: 75 mg twice daily • Weight <11 kg: Not recommended
Ritonavir (Norvir, AbbVie, Inc., “r” when used as boosting agent or RTV) **Formulation:** 100 mg tablets 100 mg packets 80 mg/mL oral solution	100 mg by mouth with each dose of DRV	**Administration:** Take with food • Tablet: Swallow tablet whole; do not chew, break, or crush • Oral solution: Mix with 8 oz of chocolate milk or Ensure and administer within 1 hour; shake well before use **Most common side effects:** Abdominal pain, weakness, headache, malaise, anorexia, diarrhea, dyspepsia, nausea, vomiting, paresthesia, dizziness, taste perversion **Drug interactions:** Screen for drug interactions^†^ **Contraindications:** Inhibits CYP3A enzyme resulting in many contraindications with medications, drug interaction review is critical **Cautions:** Can cause hepatotoxicity, pancreatitis, or hyperglycemia	**Renal:** Use standard dose **Hepatic:** Use standard dose **Pregnancy:** 100 mg of RTV by mouth with each dose of DRV (DRV 600 mg/RTV100 mg twice daily by mouth) **Pediatric:** For use as a boosting agent with darunavir (see DRV entry for RTV dosing)
Tenofovir alafenamide (Vemlidy, Gilead Sciences, Inc., TAF) **Formulation:** 25 mg tablet	25 mg once daily by mouth	**Administration:** Take with food **Most common side effects:** Headache, cough, fatigue **Drug interactions:** Screen for drug interactions^†^ **Contraindications:** None **Cautions:** Potential exacerbation of HBV infection upon initiation or discontinuation of treatment	**Renal:** eCrCl ≥15 mL/min: • Use standard dose eCrCl <15 mL/min and not on HD: • Not recommended On HD: • Use standard dose; administer after HD on dialysis days **Hepatic:** Child-Pugh class A: • Use standard dose Child-Pugh class B and C: • Not recommended **Pregnancy:** Use standard dose **Pediatric:** Aged ≥12 yrs: • Use standard dose
Tenofovir disoproxil fumarate (Viread, Gilead Sciences, Inc., TDF) **Viread formulation:** 300 mg tablet 250 mg tablet 200 mg tablet 150 mg tablet 40 mg/g oral powder **Generic formulation:** 300 mg tablet	300 mg once daily by mouth	**Administration:** Take with or without food • Oral powder: Mix with 2–4 oz of soft food using the manufacturer provided scoop, administer immediately; do not mix with liquids **Most common side effects:** Weakness, headache, diarrhea, nausea, vomiting **Drug interactions:** Screen for drug interactions^†^ **Contraindications:** None **Cautions:** Potential exacerbation of HBV infection upon initiation or discontinuation of therapy, new onset renal impairment	**Renal:** eCrCl >50 mL/min: • Use standard dose eCrCl 30–49 mL/min: • 300 mg every 48 hours eCrCl 10–29 mL/min: • 300 mg twice weekly (every 72–96 hours) eCrCl <10 and not on HD: • Not recommended On HD: • 300 mg every 7 days **Hepatic:** Use standard dose **Pregnancy:** Use standard dose **Pediatric:** Aged 2–11 yrs, weight-based dosing, tablet: • Weight ≥35 kg: 300 mg tablet once daily • Weight 28 to <35 kg:250 mg tablet once daily • Weight 22 to <28 kg:200 mg tablet once daily • Weight 17 to <22 kg: 150 mg tablet once daily • Weight <17 kg: Not recommended Aged 2–11 yrs, weight-based dosing, oral (powder): • 8 mg/kg body weight (not to exceed adult dose [300 mg once daily])
Zidovudine (Retrovir, ViiV Healthcare, ZDV)^†,§^ **Formulation:** 300 mg tablet 100 mg capsule 10 mg/mL oral syrup 10 mg/mL IV infusion (available as 20 mL single-use vial)	Not recommended for nPEP in adults (see Tables 4 and 5 for recommendations)	**Administration:** Take with or without food • Oral syrup: Measure syrup to 0.1 mL accuracy for neonates • IV administration: See package insert **Most common side effects:** Nausea, vomiting, headache, insomnia, fatigue, fever, cough **Drug interactions:** Screen for drug interactions^†^ **Contraindications:** None **Cautions:** Hematologic toxicity including anemia, neutropenia, and bone marrow toxicity	**Pediatric:** Weight ≥30 kg: • Not recommended for nPEP Aged ≥35 weeks post-conception and ≥4 weeks postdelivery, weight-based dosing using either syrup or capsule: Weight 9 to <30 kg: • 9 mg/kg twice daily by mouth Weight 4 to <9 kg: • 12 mg/kg twice daily by mouth Weight <4 kg: • Not recommended

**Abbreviations:** BIC/FTC/TAF = bictegravir/emtricitabine/tenofovir alafenamide; 3TC = lamivudine; COBI = cobicistat; DRV = darunavir; DRB/COBI = darunavir/cobicistat; DRV/COBI/FTC/TAF = darunavir/cobicistat/emtricitabine/tenofovir alafenamide; DTG = dolutegravir; eCrCl = estimated creatinine clearance calculated by the Cockcroft-Gault formula [(140 − age) x ideal body weight] ÷ (serum creatinine x 72) (x 0.85 for females); FTC = emtricitabine; FTC/TAF = emtricitabine/tenofovir alafenamide; FTC/TDF = emtricitabine/tenofovir disoproxil fumarate; HBV = hepatitis B virus; HD = hemodialysis; IV = intravenous; LPV/r = lopinavir/ritonavir; nPEP = nonoccupational postexposure prophylaxis; RAL = raltegravir; RTV = ritonavir; TAF = tenofovir alafenamide; TDF = tenofovir disoproxil fumarate; TDF/3TC = tenofovir disoproxil fumarate/lamivudine; ZDC = zidovudine.

* All listed medications are off-label use for nPEP.

^†^ Checking for drug–drug interactions is recommended for both prescription and over-the-counter products by using an interactive web-based resource such as the one from the University of Liverpool at https://www.hiv-druginteractions.org/ or https://clinicalinfo.hiv.gov/en/guidelines/hiv-clinical-guidelines-adult-and-adolescent-arv/drug-interactions-overview?view=full.

^§^ For more detailed drug information and dosing, please see the manufacturer’s package insert or consult the Guidelines for the Use of Antiretroviral Agents in Adults and Adolescents with HIV (https://clinicalinfo.hiv.gov/en/guidelines/hiv-clinical-guidelines-adult-and-adolescent-arv/whats-new).

#### Recommendations for HIV nPEP Regimens

- Complete a clinical assessment before prescribing nPEP, including assessing for medical comorbidities, current medications, and allergies (**good practice statement, standard of care**).
- The recommended nPEP course is 28 days (**good practice statement, existing recommendation**).
- The preferred regimens for adults and adolescents without contraindications are
  º bictegravir (BIC)/emtricitabine (FTC)/tenofovir alafenamide (TAF) (**recommendation, very low certainty of evidence**) OR
  º dolutegravir (DTG) plus (tenofovir alafenamide [TAF]) OR tenofovir disoproxil fumarate [TDF]) plus (emtricitabine [FTC] OR lamivudine [3TC]) (**recommendation, very low certainty of evidence**).
- Selection of a regimen should be individualized based on comorbid conditions (e.g., renal or hepatic dysfunction), pregnancy, drug interaction potential with concurrent medications, previous exposure to ARV regimens (including long-acting injectable ARV exposure), the source’s history, and regimen factors that might influence continuation of treatment (e.g., pill burden, dosing frequency, side effects, cost, and access) (**good practice statement, standard of care**).

#### Rationale for HIV nPEP Regimens

No randomized, placebo-controlled clinical trial of nPEP efficacy has been performed. A limited number of studies have evaluated the penetration of ARVs into genital tract secretions and tissues, although data are insufficient to define any ARV regimen as most effective for HIV prevention (*33*–*36*). Data relevant to nPEP recommendations are available from animal studies (*37*–*40*), prospective open-label randomized and nonrandomized experimental studies, longitudinal and observational cohort studies, case studies of nPEP use, and HIV treatment clinical trials (Supplementary Appendix A: Recommendation Strength & Rationale and Supplementary Appendix B: GRADE Tables, https://stacks.cdc.gov/view/cdc/177225#tabs-3). Regimens selected for nPEP guidelines are based on best available evidence at the time of guidelines publication; newer regimens that might become recommended for initial HIV treatment in the future also might be effective for nPEP (Appendix B).

The general recommendation for a 3-drug nPEP regimen is based on extrapolation of data demonstrating that the maximal suppression of viral replication among persons with HIV occurs when combination ARV therapy with ≥3 drugs is provided as initial therapy (*41*). Also, the recommended 3-drug nPEP regimens are expected to provide greater likelihood of protection against acquisition of resistant HIV compared with a 2-drug regimen (e.g., TDF/FTC) (*42*–*44*). In addition, the finite duration of nPEP means that the risk for cumulative toxicity of 3-drug regimens is small (*20*,*45*,*46*). Although it is included as a possible initial HIV treatment regimen in current adult HIV treatment guidelines, the 2-drug combination DTG/3TC is not a recommended nPEP regimen because of the caveats related to its use as initial treatment (e.g., not used when HIV RNA >500,000 copies/mL; not used before HIV genotypic resistance testing is available) and because no data are available related to its use for nPEP (*47*). Recommending a 3-drug regimen for all patients who receive nPEP will increase the likelihood of successful prophylaxis considering potential exposure to virus with resistance mutations (*8*). In addition, if infection occurs despite nPEP, a 3-drug regimen is more likely to limit emergence of resistance than a 2-drug regimen. Certain health care professionals have prescribed 2-drug regimens for nPEP (e.g., TDF plus FTC) in certain circumstances, such as concerns of discontinuation of treatment, toxicity, or access to medications. Data are insufficient to support a general recommendation for 2-drug regimens (see Future Research).

#### Preferred and Alternative HIV nPEP Regimens

Preferred nPEP regimens for both children aged ≥2 years and adults contain two nucleoside reverse transcriptase inhibitors (NRTIs) combined with a second-generation integrase strand transfer inhibitor (INSTI) (e.g., bictegravir or dolutegravir). Antiviral efficacy, patient tolerance and acceptability, and access were considered in selection of the regimens listed (Tables 4 and 5). In certain circumstances, health care professionals may consider using ARV regimens for nPEP other than those listed (e.g., in unique patient circumstances such as an exposure source with known drug resistance or patient contraindications to one or more ARVs). In those cases, health care professionals are encouraged to seek consultation with other clinicians knowledgeable in using ARV medications for similar patients (e.g., children, pregnant women, and persons with comorbid conditions).

Clinicians can use local resources or consult the NCCC PEPline at 888-448-4911 or https://nccc.ucsf.edu/clinician-consultation/pep-post-exposure-prophylaxis. If consultation or preferred regimens are not immediately available, any 3-drug regimen suitable for initial treatment of HIV could be used, provided it does not require pretesting (e.g., abacavir) and is not contraindicated (*47*). Although a raltegravir-based regimen is not listed because of having a higher pill burden and thus potential for discontinuation of treatment, it remains an effective option for nPEP if neither preferred nor alternative regimens can be prescribed.

#### Selecting an Initial HIV nPEP Regimen

Selection of an initial HIV nPEP regimen should be individualized. The selection should be based on comorbid conditions (e.g., renal or hepatic dysfunction), pregnancy, drug interaction potential with concurrent medications, previous exposure to ARV regimens (including long-acting injectable), source’s history (e.g., drug-resistant virus), and regimen factors (e.g., pill burden, dosing frequency, side effects, cost, and access) that facilitate adherence.

##### Presence of Certain Conditions

Certain comorbid conditions and co-infections might have a direct impact on the choice of nPEP regimen, dosing, and degree of monitoring required (Tables 5 and 6). Exposed persons who have impaired renal function might require dose adjustments of ART medications used for PEP and might require additional creatinine monitoring while completing a 28-day course of PEP. For example, TDF, when used for treatment of HIV infection, has been associated with proximal renal tubulopathy and higher rates of renal dysfunction, whereas TAF has less impact on renal function. These adverse events are less common with PEP because of its short duration (*48*). The risk for reduced tolerability and other toxicities associated with alternative regimens (e.g., zidovudine with lamivudine) must be considered, especially if there is a plan for PrEP after completion of nPEP (*49*). Liver disease with cirrhosis might be a contraindication for certain ARV regimens or might require ARV dosage modifications in persons with Child-Pugh class B or C disease (*47*). If co-infection with hepatitis B virus (HBV) is present, regimens that contain agents that treat HBV might be preferred (e.g., tenofovir and 3TC/FTC-containing regimens) (*50*,*51*).

Additional monitoring is required for exposed persons who have HBV infection, especially when stopping agents that are active in treatment for HBV infection (*47*). Elevation in liver transaminase level can occur when taking or after discontinuing ARVs and might be more common in persons with HBV or hepatitis C virus (HCV) infection (*52*). Drug-induced liver injury is more common in patients with HCV/HIV co-infection (*47*,*53*). In cases of HBV/HCV co-infection, treatment with direct-acting antiviral (DAA) agents for chronic HCV have been reported to reactivate HBV (*54*). For persons with HBV/HCV, on DAAs, or both, health care professionals are encouraged to seek consultation with other clinicians knowledgeable in using ARV medications for similar patients. Other comorbid conditions that should be considered include osteoporosis or other conditions associated with bone mineral density loss, cardiovascular disease, psychiatric illness, and substance use disorder requiring narcotic replacement (*47*). For more information regarding specific clinical scenarios, see the table “Antiretroviral Regimen Considerations for Initial Therapy Based on Specific Clinical Scenarios” of the NIH’s Guidelines for the Use of Antiretroviral Agents in Adults and Adolescents with HIV (https://clinicalinfo.hiv.gov/sites/default/files/guidelines/documents/adult-adolescent-arv/tables-adult-adolescent-arv.pdf).

##### General Considerations for Pregnant or Breastfeeding Women and Women of Childbearing Potential

Pregnancy and breastfeeding are not contraindications for nPEP. Pregnant or breastfeeding women should have rapid access to nPEP when indicated. Because of the additional considerations regarding fetal and infant safety, expert consultation might be beneficial when nPEP is prescribed to women who are pregnant or breastfeeding.

A pregnancy test should be performed in women of childbearing potential at the initial evaluation for nPEP. For women who are pregnant, consider available pregnancy safety and outcome data, known adverse effects, and pharmacokinetic properties of ARVs. Risks for adverse effects of ARVs to pregnant women and their fetuses or infants must be weighed against the risks for maternal HIV acquisition and subsequent potential perinatal HIV transmission. The appendix “Safety and Toxicity of Individual Antiretroviral Agents in Pregnancy” of the HHS Recommendations for the Use of Antiretroviral Drugs During Pregnancy and Interventions to Reduce Perinatal HIV Transmission in the United States might be useful for health care professional review and counseling of pregnant women (https://clinicalinfo.hiv.gov/sites/default/files/guidelines/documents/perinatal-hiv/guidelines-perinatal.pdf) (*6*). Pharmacokinetic changes in pregnancy might lead to lower plasma levels of certain ARVs and might require increased doses or more frequent dosing (Tables 4 and 6). Drugs not recommended for use in pregnancy because of lack of available safety and pharmacokinetic data or possible inferior virologic efficacy include cobicistat-boosted atazanavir, darunavir, and elvitegravir (*6*). Health care professionals must recognize that safety data of ARV drugs in pregnancy might be incomplete. However, robust clinical experience with ART in pregnancy includes evidence-based national guidelines recommending ART for all women with HIV who are pregnant (*6*). Additional information about ARV use in pregnant women is available (https://clinicalinfo.hiv.gov/sites/default/files/guidelines/documents/perinatal-hiv/guidelines-perinatal.pdf) (*6*).

Providing nPEP to women who are breastfeeding and are at substantial risk for HIV acquisition because of recent exposure reduces the risk for HIV acquisition and possible subsequent HIV transmission to the breastfeeding infant. Women who are breastfeeding should be counseled on the risk for HIV transmission through breastmilk should acute HIV infection occur. To eliminate any risk for HIV transmission to infants, HIV-exposed breastfeeding women might decide to stop breastfeeding. Other women who are breastfeeding might choose other courses of action (e.g., pumping and storing breastmilk until HIV infection is excluded, and then resuming the previous breastfeeding routine). Women who are breastfeeding also might be concerned about infant exposure to ARVs through breastmilk. The “Safety of Antiretroviral Drugs During Breastfeeding” portion of the “Infant Feeding for Individuals with HIV in the United States” section of the HHS Recommendations for the Use of Antiretroviral Drugs During Pregnancy and Interventions to Reduce Perinatal HIV Transmission in the United States provides an overview of the varying penetration of different ARVs into breastmilk and is a useful resource to support counseling of women who are breastfeeding prescribed nPEP (https://clinicalinfo.hiv.gov/sites/default/files/guidelines/documents/perinatal-hiv/guidelines-perinatal.pdf) (*6*). Quality counseling supports shared decision-making regarding infant feeding during an nPEP course for a mother who is breastfeeding. Health care professionals with questions about medical decision-making or counseling for women who are pregnant or breastfeeding with nPEP indications can use local consultation resources or consult the NCCC PEPline at 888-448-4911 or https://nccc.ucsf.edu/clinician-consultation/pep-post-exposure-prophylaxis or the Perinatal HIV Line at 888-448-8765 or https://nccc.ucsf.edu/clinician-consultation/perinatal-hiv-aids.

##### Drug–Drug Interactions and Medication-Related Adverse Events

Pharmacokinetic drug–drug interactions between nPEP regimens and concomitant medications are common and might lead to increased or decreased drug exposure. In certain instances, changes in drug exposure might increase toxicity frequency, severity, or both. Before prescribing nPEP, an accurate, verified medication history should be obtained, including the use of over-the-counter medications, vitamins, minerals, and herbal remedies, to identify possible drug–drug interactions. Health care professionals are encouraged to check for drug–drug interactions by using an interactive web-based resource such as from the University of Liverpool (https://www.hiv-druginteractions.org), manufacturers’ package inserts, and the NIH’s Guidelines for the Use of Antiretroviral Agents in Adults and Adolescents with HIV (https://clinicalinfo.hiv.gov/en/guidelines/hiv-clinical-guidelines-adult-and-adolescent-arv/whats-new).

Products that contain polyvalent cations (aluminum, calcium, iron, and magnesium), such as antacids or multivitamins, can bind to INSTIs and reduce absorption of the nPEP agents. Drugs or supplements that induce or inhibit the enzyme cytochrome P450 (CYP) 3A4 or efflux transporter P-glycoprotein in the intestines might reduce or promote the absorption of other drugs. Of note, the INSTIs bictegravir and dolutegravir have mixed metabolic pathways, including both CYP3A4 and uridine diphosphate glucuronosyltransferase (UGT) 1A1 enzyme (UGT1A1). Drugs that induce or inhibit these enzymes might have variable impact on the pharmacokinetics of these agents. Therefore, multiple ARV agents are contraindicated with concomitant use of certain medications (e.g., rifabutin and rifampin). Drugs that reduce gastric acidity (e.g., proton pump inhibitors) might affect the absorption of ARV agents that require acidity for optimal absorption (e.g., rilpivirine). Drug transporters also have a role in drug–drug interaction potential. For example, dolutegravir decreases renal clearance of metformin by inhibiting organic cation transporters in renal tubular cells. Therefore, metformin dosing should be limited to 1 g by mouth per day when a person is taking dolutegravir concurrently.

Newer ARV regimens, such as those recommended for nPEP, are associated with fewer serious and intolerable adverse effects. However, adverse effects have been reported with virtually all ARV regimens. As a result, the potential benefit of nPEP must be balanced with the possibility of side effects or toxicity, considering any comorbidities. When a person has experienced treatment-limiting tolerability or toxicity issues on previous HIV pre- or postexposure prophylaxis, an alternative regimen should be prescribed depending on the availability of other medications and the etiology and severity of the adverse event. Potential side effects of ARV agents should be discussed with the PEP recipient, and, when anticipated, preemptive prescribing of agents for ameliorating side effects (e.g., prescribing antiemetics or antispasmodic for regimens including ARVs commonly associated with nausea, such as zidovudine and ritonavir) might improve PEP regimen continuity of treatment. The patient should be instructed to reach out to their health care professional if they experience ARV-related adverse effects.

##### Resistance to ARV Agents and Source’s History

If the source is known to have HIV and their treatment and testing history is available at the initial nPEP visit, then the nPEP regimen can be individualized accordingly. Expert consultation might be useful for selection of an optimal PEP regimen to which the source’s virus is unlikely to be resistant; however, awaiting expert consultation should not delay the initiation of HIV PEP. If the source’s history is unavailable, administration of the exposed person’s first dose of nPEP should not be delayed. If drug resistance information becomes available later during a course of PEP, this information should be discussed with the expert consultant for possible modification of the nPEP regimen. Resistance to newer generation INSTIs is uncommon, and INSTIs are preferred if the source is known to have HIV or if a drug-resistant virus is a concern (e.g., the source has failed multiple regimens or does not take their ART as prescribed) (*41*,*42*,*47*). For instances in which nPEP fails to prevent infection, selection of resistant virus by the ARV drugs is theoretically possible. However, because of limited literature and information on resistance testing in documented nPEP failures, the likelihood of resistance occurring is unknown.

Previous exposure to long-acting injectable cabotegravir (CAB-LA) might be a risk for the presence of INSTI resistance (*47*,*55*,*56*). The pharmacokinetic study HPTN 077 found that suboptimal levels of CAB could last up to 3 years in men and 4 years in women (*56*). When the source has detectable viremia while on long-acting injectable ART (e.g., cabotegravir) or when the exposed patient has a remote history of long-acting injectable ARV use, INSTIs might not be preferred (*47*).

#### Anticipating and Facilitating Continuity of Treatment

Observational studies have reported that continuity of treatment to nPEP regimens often is inadequate, especially among sexual assault survivors. Clinicians should consider potential barriers to continuity of treatment when selecting a nPEP regimen, assess for factors that could affect continuity of treatment (e.g., concurrent substance use disorder), and refer to any needed services (*57*). Discontinuity of nPEP might be influenced largely by the convenience and side-effect profile of the ART regimen. Alternative ARV regimens can be used if a previous ARV regimen has not been well tolerated.

Medication continuity can be facilitated by tailored approaches to the persons, which include 1) prescribing medications with fewer side effects, fewer doses per day, and fewer pills per dose; 2) educating patients regarding potential side effects of the specific ARVs prescribed and providing medications to assist if side effects occur (e.g., antiemetics); 3) recommending medication continuity aids (e.g., pill boxes and smartphone reminders); 4) helping patients incorporate doses into their daily schedules; and 5) providing a flexible and proactive means for patient–health care professional contact during the nPEP course (*58*–*60*). Also, establishing a trusting relationship and maintaining good communication about adherence can help to improve completion of the nPEP course. Adherence to the nPEP medications prescribed to children will depend on the involvement of and support provided to parents and guardians. Adherence counseling should be nonjudgmental and should highlight a review of strategies to avoid missed doses and approaches tailored to the person (e.g., incorporating the nPEP regimen into daily routines or setting smartphone reminders).

##### Cost and Access

ARV medications are expensive, and persons in need of nPEP might be unable to cover the out-of-pocket costs. Ensuring nPEP access requires a thorough assessment of costs and patients’ ability to obtain a full course of nPEP. Options exist to reduce cost. When public, privately purchased, or employer-based insurance coverage is unavailable, health care professionals can assist patients with obtaining ARV medications through the medication assistance programs of the pharmaceutical companies that manufacture the prescribed medications. Online applications are available or certain companies can be called on an established phone line. Requests for assistance often need to be handled urgently so that accessing medication is not delayed. Health care providers should also be aware that generic ARV options are available among the recommended nPEP regimens.

Pharmacy dispensing practices have sometimes been a barrier to timely nPEP access because certain pharmacies have policies requiring them to call the prescriber for ARV scripts of 28 days (because of packaging of certain ARVs in 30-day supply bottles). To overcome this barrier, health care professionals might choose to prescribe a 30-day nPEP supply with instructions to the nPEP user that the course can be considered complete after 28 days of medication. When the source is present during a patient’s nPEP evaluation, health care professionals can also assess the source’s access to relevant medical care, behavioral interventions, and social support services and provide relevant treatment, referrals, or both, as indicated.

### Laboratory Testing and nPEP Follow-Up

At nPEP initiation, laboratory testing is required to exclude pre-existing HIV infection, obtain baseline renal and liver function tests, and evaluate other conditions depending on the circumstances of the exposure to assure selection of the safest, most appropriate ARV regimen. Laboratory testing also is recommended at the end of the course to check for HIV infection. Recommendations are presented for the most appropriate tests and time of testing (Table 3).

#### Recommendations for Laboratory Testing and nPEP Follow-Up

- Persons being assessed due to a known or possible exposure to HIV should be tested for HIV (**good practice statement, existing recommendation**).
- At the initial nPEP medical visit, a rapid (also referred to as point-of-care), laboratory-based antigen/antibody combination (Ag/Ab) HIV test, or both, is recommended (**good practice statement, existing recommendation**).
- For persons with long-acting injectable PrEP ARV exposure during the past 6 months, a diagnostic HIV nucleic acid test (NAT) is recommended at the initial medical evaluation, in addition to an Ag/Ab HIV test (**good practice statement, indirect data; existing recommendation**).
- Perform interim HIV testing with both a laboratory-based HIV Ag/Ab test plus a diagnostic HIV NAT test 4–6 weeks after exposure (**good practice statement, standard of care**).
  º HIV testing 4–6 weeks post-nPEP initiation may be deferred for persons who started nPEP within 24 hours of a known or possible HIV exposure and who did not miss any nPEP doses.
- Perform final HIV tests using laboratory-based HIV Ag/Ab combination immunoassay and diagnostic HIV NAT 12 weeks after exposure (**good practice statement, standard of care**).
- Routine laboratory testing recommended for persons starting nPEP includes serum creatinine, alanine aminotransferase (ALT), and aspartate aminotransferase (AST), as well as HIV, hepatitis B virus (HBV), and pregnancy testing (**good practice statement, existing recommendation**).
- Testing and treatment of hepatitis C virus (HCV) infection, other STIs including gonorrhea, chlamydia, and syphilis, and other medical treatment should be tailored to the clinical situation (**good practice statement, existing recommendation**).

##### HIV Testing and nPEP

Laboratory testing is required to 1) document HIV status of the person seeking an nPEP evaluation (and the exposure source when available and consent has been granted), 2) identify and clinically manage any other conditions that could result from sexual or injection-related exposure to potentially contaminated body fluids, 3) identify any conditions that would affect selection of the nPEP medication regimen, and 4) monitor for safety or toxicities related to the regimen prescribed. Types of HIV tests include nucleic acid tests (NATs) that detect HIV RNA; antigen/antibody combination (Ag/Ab) tests that detect the HIV p24 antigen as well as HIV immunoglobulin M (IgM) and immunoglobulin G (IgG) antibodies; and antibody (Ab) tests that detect HIV IgM antibodies, IgG antibodies, or both. Different types of HIV tests have different window periods (time between HIV exposure and ability to detect HIV infection) and different sensitivities to detect HIV in the setting of recent ARV exposure. Resources are available to provide additional information about HIV testing (*28*,*61*).

##### HIV Testing at the Initial Visit

The goal of HIV testing at the initial nPEP encounter is to assess whether the person has HIV without delaying nPEP initiation. Persons with a recent known or possible HIV exposure should be tested for HIV using a rapid (point-of-care) or laboratory-based Ag/Ab test. If a rapid (point-of-care) test is used, then a laboratory-based Ag/Ab test is also recommended to increase the sensitivity for detecting HIV (*62*). Certain experts would include an HIV NAT in nPEP baseline testing, especially if the person has recently taken oral ARVs or had a cabotegravir injection during the past 6 months. The benefits of a diagnostic HIV NAT include increased sensitivity (compared with Ag/Ab tests) to detect HIV in the setting of ARV exposure and the shortest time to detection among available HIV tests. Potential problems associated with a diagnostic HIV NAT include access issues, notably a lack of availability in certain areas or systems and increased costs. If the recommended HIV test is not accessible, the most sensitive available test should be used. Oral fluid–based rapid HIV tests are not recommended for HIV screening in the context of nPEP services because they are less sensitive for the detection of acute or recent infection than blood tests (*62*). nPEP services should not be withheld because of a lack of availability of HIV NATs (*62*). nPEP should be initiated as soon as possible after the rapid (point-of-care) test result (if available). The initial nPEP dose should not be delayed while awaiting results of laboratory-based testing.

At the initial nPEP visit, health care professionals should inform the person being tested for HIV that the results cannot identify HIV acquisition from the recent (≤72 hours) exposure. This counseling can reinforce the importance of follow-up HIV testing. Persons being assessed after a recent HIV exposure also should be educated about the signs and symptoms associated with acute HIV infection, including fever, rash, or influenza- or mononucleosis-like symptoms, and asked to return for evaluation if these occur before the final nPEP clinical visit (at the time of final follow-up HIV testing 12 weeks after exposure). The initial nPEP visit is also an opportunity to assess ongoing risk for HIV acquisition and provide education about HIV PrEP (*5*).

Persons with a positive rapid (point-of-care) HIV test at the initial visit should receive supplemental diagnostic testing as soon as possible (*61*). If HIV infection is diagnosed in a person who is taking PEP, the PEP regimen should be continued until they are evaluated by an HIV treatment specialist. Linkage to HIV treatment should occur as soon as possible.

Persons with a recent known or possible HIV exposure who decline nPEP should still be offered baseline HIV testing and be informed that this testing cannot detect HIV acquisition from the recent (≤72 hours) exposure. Counseling should be provided about the signs and symptoms of acute HIV infection, the increased risk for HIV transmission during acute HIV infection, and the importance of follow-up testing (*63*,*64*). Follow-up HIV testing with a laboratory-based Ag/Ab test is recommended 6 weeks after exposure for persons who decline nPEP.

##### HIV Testing at Follow-up Visits

ARVs taken as PEP and PrEP can suppress HIV viral load, delay seroconversion, and decrease the ability to detect HIV infection. To improve the likelihood of diagnosing HIV infection among persons who have recently taken PEP, both a laboratory-based Ag/Ab test and diagnostic NAT are recommended as follow-up testing (*65*–*67*). Inability to provide HIV NATs should not prevent provision of nPEP to persons with indications. Health care professionals should use the most sensitive accessible HIV test if the recommended test is not available. Oral fluid–based rapid HIV tests are not recommended for HIV screening in the context of nPEP services because they are less sensitive for the detection of acute or recent infection than blood tests (*62*).

The first follow-up test with both a laboratory-based Ag/Ab test and a diagnostic NAT can be performed 4–6 weeks after nPEP initiation (i.e., within 2 weeks of completion of the full course of nPEP). Testing at this time might identify HIV, particularly in persons who did not adhere to the nPEP regimen or did not complete the 28-day course. A negative test at this time does not rule out HIV infection because ARVs provided for nPEP might suppress HIV for longer than 2 weeks after stopping the medications. A follow-up visit with HIV testing 4–6 weeks after PEP initiation is also an appropriate time to assess for PrEP indications and to start PrEP if indicated and desired (*5*). Although HIV testing 4–6 weeks post-nPEP initiation is preferrable, HIV testing 4–6 weeks post-nPEP initiation can be deferred for persons who started nPEP within 24 hours of a known or possible HIV exposure, were adherent to the complete nPEP course, and are not considering starting PrEP.

Persons initiating PrEP before the final follow-up HIV testing 12 weeks after PEP initiation should be counseled about the possibility of a false-negative HIV test result and the importance of ongoing PrEP care with recommended HIV testing. Available data indicate that nPEP is highly effective when taken as prescribed, and PrEP guidelines do not recommend a gap between nPEP conclusion and PrEP initiation (*5*).

The final follow-up test, with the purpose of ruling out HIV infection, should include both a laboratory-based Ag/Ab test and a diagnostic NAT 12 weeks after PEP initiation (8 weeks after PEP completion). This timing is recommended based on data about the timeline for ARV washout and the window period of the HIV tests (*68*,*69*). Most laboratory-based Ag/Ab tests should be able to detect HIV acquisition from the initial exposure; however, certain observational studies have demonstrated nPEP failures attributable to subsequent exposures (which might not be disclosed) (*24*). Diagnostic NATs can detect acute HIV infection approximately 1 week before laboratory-based Ag/Ab tests, improving the likelihood of accurately diagnosing HIV at a time when a person is highly infectious (*68*). For persons who have not yet started PrEP, indications and interest in PrEP for HIV should be reassessed at this time (*5*).

If 12-week follow-up testing is not obtained, then HIV testing should be performed as soon as possible and prioritized at the next health care visit. Screening for PrEP indications and interest should also be done at this time.

#### Other Laboratory Studies

To guide the selection of an appropriate ARV regimen for nPEP, all patients who will be prescribed nPEP should have serum creatinine measured, an estimated creatinine clearance computed, and serum ALT and AST measured as well. HBV testing at nPEP initiation is indicated because tenofovir (both TAF and TDF) and 3TC and FTC are active against HBV and abrupt withdrawal of medications active against hepatitis B can lead to a hepatitis B flare. Initiation of nPEP should not be delayed while waiting for laboratory test results. If needed, the nPEP regimen can be modified after the first dose once laboratory test results are received. Routine follow-up testing of serum creatinine, AST, and ALT is not necessary unless baseline tests are abnormal or clinical indications are present (e.g., signs and symptoms concerning for kidney or liver injury). A pregnancy test should be done for all women with childbearing potential who are evaluated for nPEP.

Newer ART regimens have fewer side effects and are better tolerated than earlier regimens (*20*,*70*). Multiple studies support the safety of tenofovir-containing regimens in both persons with and without HIV (*71*–*74*). Small declines in renal function might occur with daily tenofovir; however, these reverse upon cessation and the incidence of serious renal events is very low because of PEP’s short-term duration. The potential for adverse renal events is lower with TAF than with TDF (*48*). Elevations in liver transaminase level can occur when taking or after discontinuing ARVs and might be more common in persons with hepatitis B or hepatitis C. These hepatic side effects are less prevalent with integrase inhibitors than with protease inhibitors (*70*). Elevations in lipid levels can occur when taking TAF (*75*). Closer monitoring is recommended if new signs and symptoms develop while taking nPEP (e.g., rash, jaundice, and muscle pain), if the recipient is pregnant, if there is a risk for drug–drug interaction, if substantial comorbidities such as hepatitis or renal dysfunction exist, or if significant abnormalities on baseline testing are detected. If muscular soreness develops while taking PEP, particularly INSTI-based PEP, creatinine kinase should be checked. More information about laboratory testing in the setting of ARV use is available in the “Laboratory Testing” section of the NIH’s Guidelines for the Use of Antiretroviral Agents in Adults and Adolescents with HIV (https://clinicalinfo.hiv.gov/en/guidelines/hiv-clinical-guidelines-adult-and-adolescent-arv/tests-initial-assessment-follow-up?view=full).

#### STI Testing, PEP, and Presumptive Treatment

Any sexual exposure that presents a risk for HIV infection also might place a person at risk for acquiring other STIs. CDC STI Treatment Guidelines, 2021, recommend presumptive STI treatment after sexual assault because clinical follow-up often is challenging for survivors (*76*). Presumptive STI treatment and PEP must be tailored to the clinical situation and might include an empiric antimicrobial regimen effective against chlamydia, gonorrhea, and trichomonas for women and chlamydia and gonorrhea for men; postexposure hepatitis B vaccination with or without hepatitis B immunoglobulin (as indicated by the hepatitis B immune status of the exposed person and the hepatitis B infection status of the source); and human papillomavirus or mpox vaccination (see relevant guidelines for specific indications) (*76*–*78*). Certain health care professionals, in shared decision-making with a sexual assault survivor, might await STI test results rather than provide presumptive STI treatment. If the initial STI tests are negative and presumptive STI treatment was not provided, STI testing can be repeated 1–2 weeks after the exposure (*76*,*78*). For GBMSM, a single 200 mg dose of doxycycline taken within 72 hours of condomless sex (doxycycline postexposure prophylaxis, or “doxy-PEP”) might be considered as part of a comprehensive approach to STI care (*79*). Health care professionals who provide nPEP should remain up to date with relevant guidelines for STI diagnosis and treatment (*77*–*81*).

#### nPEP Follow-Up and Counseling

Follow-up care is necessary for patients prescribed nPEP medications to monitor for adverse effects, follow up laboratory testing, support adherence, and optimize HIV prevention strategies (e.g., transitioning to PrEP when indicated). Before the person leaves the initial nPEP encounter, a plan for the recommended follow-up visits and testing should be in place, with appropriate referrals and resources provided. The health care professional who provided or prescribed the nPEP medications, or a support staff member, should follow up with the person within 24 hours to confirm access to the medications and assess tolerability and adherence. If the person does not tolerate the recommended regimen, the health care professional should consider switching to an alternative regimen to improve continuation of treatment, and consultation might be useful to troubleshoot issues with the initial regimen. Local resources are available in many places, and nationally the NCCC PEPline (888-448-4911 or https://nccc.ucsf.edu/clinician-consultation/pep-post-exposure-prophylaxis) is available.

Health care professionals providing nPEP services should be aware that unusual or severe toxicities from ARV medications can be reported to the manufacturer or the FDA (https://www.accessdata.fda.gov/scripts/medwatch/medwatch-online.htm or 1-800-FDA-1088 [1-800-332-1088]). If nPEP is prescribed to a woman who is pregnant at the time of exposure or becomes pregnant while taking nPEP, health care professionals can contribute clinical information into the Antiretroviral Pregnancy Registry (https://www.apregistry.com/).

 Clinical considerations include providing counseling to persons staring nPEP (see Clinical Considerations when Starting nPEP). Topics to cover include ways to reduce the risk for transmitting HIV if acquired during the nPEP period (e.g., avoiding condomless sex, avoiding sharing drug injection equipment, and not donating blood or tissues until the guideline criteria for donation have been met) (*82*).

### Transitioning to PrEP After PEP

Persons who receive nPEP might have continuing exposures that put them at ongoing risk for acquiring HIV. These persons might benefit from HIV PrEP to reduce their risk for acquiring HIV infection. When taken as prescribed, PrEP can reduce the risk for acquiring HIV from sex by about 99% and from injection drug use by at least 74% (*20*,*74*,*83*–*88*).

#### Recommendation for Transitioning to PrEP After PEP

- An immediate transition from nPEP to PrEP, including HIV testing at the completion of the nPEP regimen with a prompt transition to a recommended PrEP regimen, might be beneficial for persons with anticipated repeat or ongoing potential HIV exposures (**good practice statement, existing recommendation**).

#### Rationale for Transitioning to PrEP After PEP

Studies that report on HIV incidence after a complete course of nPEP are not always able to determine whether seroconversion was because of nPEP failure or subsequent re-exposure. However, multiple observational studies have reported 0.37%–9% of persons who have taken nPEP acquired HIV infection after nPEP completion (*4*,*68*,*69*,*87*–*89*).

HIV incidence in nPEP users can be at least partially attributed to ongoing exposure risk. In a retrospective cohort study of nPEP seekers in an outpatient HIV clinic in Poland, 12% (12 of 98) of persons who took PEP for sexual exposure continued the same pattern of exposure after PEP completion (*90*). The median time to next exposure was 1.55 (IQR = 0.78–2.43) months with risk for having another exposure increasing with age and for GBMSM (*87*). In a retrospective data linkage study of HIV incidence among nPEP seekers at a large tertiary care hospital in Switzerland, the HIV incidence rate among GBMSM who sought nPEP was 70.5 per 10,000, almost twice the overall incidence among GBMSM in Zurich (39 per 10,000). The rate among GBMSM with more than one nPEP course was even higher (81.1). The median time between the last nPEP consultation and HIV diagnosis was 4.1 (IQR = 2.3–6.4) years (*88*).

All persons prescribed nPEP should be assessed for ongoing risk for HIV acquisition. For persons who might benefit from PrEP, initiation can occur at any time after nPEP completion (*89*). Available data indicate that nPEP is highly effective when taken as prescribed and all persons prescribed nPEP should be assessed for ongoing risk for HIV acquisition. Available data indicate that nPEP is highly effective when taken as prescribed and PrEP guidelines do not recommend a gap between nPEP conclusion and PrEP initiation (*89*). ARVs can delay HIV diagnosis if HIV acquisition occurred during the exposure preceding nPEP (*5*,*68*). Persons initiating PrEP before the final follow-up HIV test 12 weeks after PEP initiation should be counseled about the possibility of a false-negative HIV test result and the importance of ongoing PrEP care with recommended HIV testing. Additional information about HIV testing is available (see Laboratory Testing and nPEP Follow-Up). Further information on transition from nPEP to PrEP is available in the “Nonoccupational Postexposure Prophylaxis” section of the most recent CDC PrEP Guidelines (*5*).

## CDC Guidance

### Clinical Considerations when Starting nPEP

The following section provides clinical guidance when implementing nPEP evaluation and therapy. During the initial encounter, the health care professional should use a culturally competent, trauma-informed approach with clear and direct language, avoiding language that could be potentially shaming or stigmatizing (*90*). The trauma-informed approach to care realizes the impact of trauma, recognizes how trauma can affect all persons involved in the situation, and responds to this knowledge by putting policies and practices in place that facilitate avoidance of retraumatization (*91*). The health care professional should provide information that is tailored to the person’s health literacy level (*90*,*92*). Clinicians should consider many factors when starting someone on nPEP.

#### Implementation Considerations when Starting nPEP

- Health care professionals should ensure the first dose of nPEP is provided as soon as possible, during the clinical encounter if feasible. Alternatively, the prescriber or a support staff member should ensure a plan for timely prescription fulfillment after the clinical encounter.
- Health care professionals should use a culturally respectful, trauma-informed approach to provide nPEP and the associated tests and counseling.
- Persons being assessed for nPEP should receive all medically indicated care, including treatment of wounds and other conditions at presentation, and screening for safety, mental health concerns, substance use disorder, pregnancy, STIs, and other conditions indicated by the clinical presentation.
- Health care professionals providing nPEP for children and adolescents should be aware of reporting requirements for child abuse and legal issues about consent for clinical care. Expert consultation might be helpful to ensure the unique needs of children and adolescents are addressed.
- The clinical team should ensure that client-tailored nPEP education is provided and that a clear plan is developed for nPEP completion, follow-up and testing, and transition to either PrEP or routine care. Development of a follow-up plan includes screening and problem-solving about potential barriers to care (e.g., medication costs, transportation difficulties, and others).
- HIV nPEP should be offered to survivors of sexual assault as part of comprehensive post-assault services when the assault included any contact associated with substantial risk for HIV transmission and the source is known to have HIV or the source’s HIV status is unknown.
- Women who are pregnant or breastfeeding should be offered nPEP when indicated. Because of added considerations for counseling and ARV prescribing in women who are pregnant or breastfeeding, expert consultation might be helpful. Consultation could occur with local resources, or the National Clinician Consultation Center PEPline (888-448-4911 or https://nccc.ucsf.edu/clinician-consultation/pep-post-exposure-prophylaxis).
- For women who are breastfeeding their infant and who potentially have been exposed, evidence-based, patient-centered counseling should support shared decision-making about infant feeding. This counseling should include a discussion about the risks for and benefits of continuing versus interrupting breastfeeding while taking nPEP and being monitored for HIV acquisition.
- The initial nPEP dose should not be delayed due to pending results of any laboratory-based testing.
- If the recommended HIV test is not available, the most readily accessible HIV test with the highest sensitivity should be used. nPEP services should not be withheld if an HIV NAT is not available.
- Oral fluid–based HIV tests are not recommended for HIV screening in the setting of nPEP.
- If a rapid (point-of-care) test is used, a parallel laboratory-based Ag/Ab test also should be performed to increase the sensitivity for detecting HIV.
- Routine follow-up testing of serum creatinine, AST, and ALT is not necessary unless baseline tests are abnormal or clinical indications are present (e.g., signs and symptoms concerning for kidney or liver injury).
- The health care professional who prescribed nPEP, or a support staff member, should follow up with the person prescribed nPEP within 24 hours of the initial visit to confirm access to the medications and to assess initial tolerability and adherence.
- Medical follow-up for persons prescribed nPEP should be tailored to the clinical situation and should include at minimum a visit at 24 hours (remote or in person) with a clinical care provider and clinical follow-up 4–6 weeks and 12 weeks after exposure for laboratory testing. At this visit, providers should address any barriers to obtaining nPEP medications, access for medication side effects, and provide any other clinically indicated care (see Counseling and Education).
- Persons initiating nPEP should be informed that PrEP can reduce their risk for acquiring HIV if they will have repeat or continuing exposure to HIV after the end of the nPEP course.
- Health care professionals should offer PrEP options to persons with ongoing indications for PrEP and create an nPEP-to-PrEP transition plan for persons who accept PrEP.
- Persons initiating PrEP before the final follow-up HIV test 12 weeks after PEP initiation should be counseled about the possibility of a false-negative HIV test result and the importance of ongoing PrEP care with recommended HIV testing.
- An immediate transition from nPEP to PrEP, including HIV testing at the completion of the nPEP regimen with a prompt transition to a recommended PrEP regimen, might be beneficial for persons with anticipated repeat or ongoing potential HIV exposures, especially for persons who might be exposed to HIV during the time between completion of the nPEP course and the recommended HIV test 12 weeks after exposure.

After assessing for nPEP indications, the health care professional should discuss starting nPEP. If nPEP is indicated and the person agrees to start, the first dose should be administered as soon as possible. If the person does not agree to start nPEP, then reasons for declining might be explored and addressed if possible. If nPEP is declined, the decision should be documented in the medical record and guidance should be provided about returning for nPEP as soon as possible and within 72 hours of the exposure if the person changes their mind. In addition, information and referrals should be provided about PrEP and other indicated preventive care and harm reduction services. Persons seeking nPEP services should be screened for sexual assault, domestic violence, abuse, trafficking, suicidal ideation, and other safety concerns, as applicable. Screening for mental health and substance use disorders might be useful when resources, referrals, or other interventions are available to address identified issues. Resources are available to guide effective screening for safety issues, suicidal ideation, and mental health and substance use disorders (*93*–*95*). Persons seeking nPEP services should be provided standard of care for any injuries, wounds, or other medical needs at the time of clinical presentation.

#### Medication Provision and Testing

The first dose of nPEP should be administered as soon as possible during the initial encounter. The initial nPEP dose should not be delayed due to pending results of any laboratory-based testing. If the recommended HIV test is not available, the most readily accessible HIV test with the highest sensitivity should be used. If a rapid (point-of-care) test is used, then a parallel laboratory-based Ag/Ab test also should be performed to increase the sensitivity for detecting HIV. nPEP services should not be withheld if an HIV NAT is not available. Oral fluid–based HIV tests are not recommended for HIV screening in the setting of nPEP. Routine follow-up testing of serum creatinine, AST, and ALT is not necessary unless baseline tests are abnormal or clinical indications are present (e.g., signs and symptoms concerning for kidney or liver injury).

After the initial dose is administered, the options for medication provision are to provide the rest of the full 28-day supply of medication; to provide a starter pack with typically a 3- to 5-day supply of medication and plan for how to obtain the rest of the course; or to provide a prescription for the rest of the course. If a prescription is provided, the health care professional should counsel the person on filling the prescription and starting the medication as soon as possible and the importance of adherence to the daily regimen and taking the full course. Every effort should be made to ensure that the person receives and takes the full course as prescribed. Adherence support tools through smartphone apps, calendar reminders, pill containers, or taking the pill as part of a daily routine might be helpful.

Certain studies have found that starter packs might be associated with lower completion rates (*96*). Starter pack provision might be an option in certain settings because the completion of the nPEP course was high (77%) among GBMSM at sexual health clinics in New York City who were administered starter packs (*97*). However, among persons provided starter packs from emergency departments, attendance at follow-up appointments was low (38%–47%) with associated lower nPEP completion rates (*98*,*99*). For persons provided a starter pack supply of medication, a prescription for the rest of the course could be provided at the initial encounter or, if the person does not have any barriers to attending follow-up care, at a follow-up visit scheduled at least 1 day before the starter pack ends. Evidence is insufficient to determine the optimal approach to providing nPEP. Local jurisdictions, health care systems, organizations, and health care professionals who prescribe nPEP should develop protocols for nPEP provision that considers local factors such as follow-up options, medication availability, and other social and structural considerations so that all health care professionals are supported to provide nPEP as recommended to all persons who would benefit from this intervention.

#### Counseling and Education

Health care professionals should ensure that persons seeking nPEP services are provided with necessary counseling and education about nPEP and HIV prevention. The health care professional who prescribed nPEP, or a support staff member, should follow up with the person prescribed nPEP within 24 hours of the initial visit to confirm access to the medications and to assess initial tolerability and adherence. Medical follow-up for persons prescribed nPEP should be tailored to the clinical situation and should include at minimum a visit at 24 hours (remote or in person) with a medical provider, and clinical follow-up 4–6 weeks and 12 weeks after exposure for laboratory testing. Resources, including health care professional checklists, are available (*14*). Counseling should include the following topics:

- The likelihood that the person was exposed to HIV and their risk for infection
- Indication and timeline for nPEP initiation
- Importance of taking the first dose as soon as possible, taking the medication daily, and taking the full course unless instructed to stop by a health care professional
- Purpose of initial HIV test and interpretation of results
- Purpose of other initial laboratory tests
- What will happen if the exposed person’s HIV test is positive
- What will happen if the source’s HIV status becomes known
- Plan for and importance of follow-up visit and HIV testing
- Possible drug interactions based on the person’s current medications and supplements
- How and when to take the PEP medications
- How to get the PEP medications if the full 28-day course is not provided
- Possible adverse effects of the PEP medication and what to do if they occur
- What to do if a dose of PEP is missed
- Recognition of signs and symptoms of acute HIV infection and what to do if they occur
  º Signs and symptoms of acute HIV infection might include influenza- or mononucleosis-like illness, fever, night sweats, swollen or enlarged lymph nodes, muscle or joint pains, sore throat, feeling tired or ill, headache, rashes, or sores. If any of these occur in the months after the potential HIV exposure, the person should seek medical attention and request an HIV test.

Counseling also should include recommendations to reduce the risk for HIV transmission if the exposed person acquires HIV during the nPEP period. These recommendations include avoiding condomless sex, avoiding sharing drug injection equipment, and not donating blood or tissues until the guideline criteria for donation have been met (*82*).

#### Considerations for Persons who Experienced Sexual Assault

HIV nPEP should be offered to survivors of sexual assault as part of comprehensive post-assault services when the assault included any contact associated with substantial risk for HIV transmission and the source is known to have HIV infection or the source’s HIV status is unknown. Comprehensive discussion of the care of persons who have experienced sexual assault and abuse are beyond the scope of these guidelines; however, certain considerations for health care professionals are discussed. In addition, it is useful for health care professionals to be aware of local resources and published guidance, such as the American College of Obstetricians and Gynecologists (ACOG) Committee Opinion No. 777 on sexual assault and the American Academy of Pediatrics resources on caring for adolescents and children after sexual assault and abuse (*93*,*100*,*101*).

Multiple reports from the United States suggest that many sexual assault survivors struggle to complete nPEP (range = 3%–76%) (*102*–*104*). In one study, only 59% of patients who were offered PEP accepted it, and of those who accepted, only 15% were known to have completed the course (*105*). The proportion of patients who experienced sexual assault who had follow-up HIV testing within 6 months of receiving a PEP prescription was 12%–20%, even when the initial evaluation occurred in an emergency department with a PEP protocol (*103*,*106*). After implementation of a multidisciplinary PEP program in a U.S. emergency department, only 11% of PEP patients attended their 4-week follow-up appointment (*107*).

Implementation of protocols and multidisciplinary programs in emergency departments that involve health care professional training, order sets and other clinical decision support tools, and support of pharmacists and sexual assault forensic or nurse examiners can improve evaluation, testing, medication provision, and follow-up (*108*,*109*). Programs that provide the first dose at the initial evaluation and provide medication in hand before discharge with either the full course or a starter pack should be considered (*110*). Health care professionals should use a culturally sensitive, empathetic, trauma-informed approach and be aware of the potential for substantial emotional distress and how this might affect a patient’s comprehension of their care plan (*108*,*109*). Factors that might improve adherence and completion include encouragement by the health care professional to take nPEP, co-located services with HIV testing and nPEP provision at initial consultation, knowledge that the perpetrator has HIV infection, transportation support, counseling, and treatment reminders (*111*).

If available, a sexual assault forensic or nurse examiner should be consulted to provide optimal care and follow-up coordination. If the person is not being seen in an emergency department for the initial encounter, the health care professional should refer the person for a forensic examination and treatment at an emergency department or sexual assault treatment center after any immediate health concerns have been addressed. The Rape, Abuse & Incest National Network maintains a national hotline to assist those affected by sexual violence, as well as an online search tool to locate sexual assault services and health care professionals (*112*). Health care professionals at the initial encounter who do not have access to these services should familiarize themselves with appropriate care for persons who have experienced sexual assault, should assess the person’s safety before discharge, and should provide referrals and resources for follow-up care and support (*93*,*112*,*113*). If a forensic examination is to occur, the person should be instructed to not change their clothes, bathe or shower, eat or drink, urinate or defecate, or douche until they have been examined, if possible; however, if they have done so, they should still be encouraged to undergo the forensic examination. The forensic examination should be performed by the most qualified health care professional available. For more information on the sexual assault medical forensic examination, health care professionals can review institutional protocols and can consult available comprehensive resources for further information (*114*).

In addition to nPEP services, health care professionals evaluating persons after sexual assault should provide all indicated medical care including treatment of injuries, provide services or referrals to address psychological trauma, and address potential for STIs, pregnancy, or both. ACOG’s Committee Opinion on sexual assault discusses counseling and contraception, and CDC has published guidelines for STI screening and treatment after sexual assault (*76*,*93*).

Certain states and localities have special programs that provide reimbursement for medical therapy after sexual assault, including ARV medication, and those areas might have specific reporting requirements. In all states, sexually assaulted persons are eligible for reimbursement of medical expenses through the U.S. Department of Justice Victim’s Compensation Program in cases where the sexual assault is reported to the police (*115*).

#### Considerations Related to Pregnancy or Breastfeeding

Pregnant or breastfeeding women should be offered nPEP when indicated. Because of added considerations for counseling and ARV prescribing during pregnancy or breastfeeding, expert consultation might be helpful. Consultation could occur with local resources or the NCCC PEPline at 888-448-4911 or https://nccc.ucsf.edu/clinician-consultation/pep-post-exposure-prophylaxis. For women who are breastfeeding their infant and exposed, evidence-based, patient-centered counseling should support shared decision-making about infant feeding. This counseling should include a discussion about the risks for and benefits of continuing versus interrupting breastfeeding while taking nPEP and being monitored for HIV acquisition. Additional information about nPEP and pregnancy is available (see HIV nPEP Regimens).

#### Considerations for Children and Adolescents

If a child or adolescent has concerns related to sexual abuse or assault, the health care professional must assess whether the child or adolescent is safe to discharge from medical care. If the child or adolescent is considered to be at imminent risk for harm, this is a child protection emergency, and authorities (child protective services and law enforcement) must be contacted immediately (*101*). The child or adolescent should remain under medical staff supervision until the contacted authorities have established a plan for further care.

When children or adolescents with concerns related to sexual abuse or assault are not considered to be at imminent risk for harm, health care professionals still must be aware that health care workers in the United States are mandated by law to report suspected child maltreatment (*101*). Health care professionals must be aware of the reporting requirements and procedures in their state or territory for reporting suspected child abuse.

Health care professionals should also be aware of local laws and regulations that govern which clinical services minors can access with or without previous parental consent. In certain jurisdictions, minors of particular ages can access contraceptive services, STI diagnosis and treatment, HIV testing, and nPEP and PrEP care without parental or guardian consent (*116*). When available, consultation with health care professionals with specific training in the care of children and adolescents, or particularly specialists in pediatric sexual assault or abuse, might facilitate care. A trauma-informed approach that avoids retraumatization and uses objective, open-ended, nonleading, developmentally appropriate language should be used. Investigation of child abuse should include a forensic interview by a specially trained professional, and repeated interviews of children should be avoided (*101*).

#### Considerations for Persons who Inject Drugs or who Have Substance Use Disorder

The preferred nPEP regimens for adults and adolescents can be taken with methadone or buprenorphine, with no dose adjustment needed. Health care professionals should consider discussing substance use disorder treatment options, assessing availability and use of safe injecting practices, providing naloxone if available, and assessing for co-occurring mental health disorders. If indicated and desired, the health care professional should refer the person for substance use disorder treatment and harm reduction programs (e.g., syringe service programs), if available. Resources are available for additional information about care for persons who inject drugs (*95*,*117*,*118*).

#### Considerations for Persons Receiving Hormone Therapies

The preferred nPEP regimens can be taken with hormone therapies (estrogens or testosterone) with no dose adjustment needed. Side effects from the interaction between certain hormone therapies and certain nPEP regimens (e.g., ritonavir and cobicistat-containing regimens) can occur and should be monitored for by health care providers (*3*,*119*).

#### Considerations for PrEP After nPEP

Persons initiating nPEP should be informed that PrEP can reduce their risk for acquiring HIV infection if they will have repeat or continuing exposure to HIV after the end of the nPEP course. Health care professionals should offer PrEP options to persons with ongoing indications for PrEP and create an nPEP-to-PrEP transition plan for persons who accept PrEP. Persons initiating PrEP before the final follow-up HIV test 12 weeks after PEP initiation should be counseled about the possibility of a false-negative HIV test result and the importance of ongoing PrEP care with recommended HIV testing. An immediate transition from nPEP to PrEP, including HIV testing at the completion of the nPEP regimen with a prompt transition to a recommended PrEP regimen, might be beneficial for persons with anticipated repeat or ongoing potential HIV exposures, especially for persons who might be exposed to HIV during the time between completion of the nPEP course and the recommended HIV test 12 weeks after exposure. Further information on transition to PrEP can be found in the most recent CDC PrEP Guidelines (*5*).

### HIV nPEP Access

The first step in nPEP use is for eligible persons to know about nPEP as an option and to seek care within 72 hours of a possible exposure; additional steps include obtaining and taking the medications appropriately. Social and structural barriers might affect a person’s ability to access, start, adhere to, and complete nPEP and to receive recommended follow-up care. These barriers might include stigma and shame, cost of the medications, difficulty with rapid access to medications, and lack of access to knowledgeable health care professionals for nPEP initiation and follow-up care (*108*,*120*,*121*).

#### Implementation Considerations for HIV nPEP Access

- Health care institutions, systems, and local health departments should have established protocols to facilitate nPEP access. nPEP access includes timely nPEP delivery, medically appropriate clinical assessment and treatment, medication access, all indicated referrals and services, completion of recommended follow-up and testing, and transition to PrEP services or continuing standard clinical care.
- Health care institutions, systems, and local health departments might benefit from considering innovative nPEP delivery strategies including nurse- and pharmacist-led approaches.
- Health care professionals might consider a “PEP-in-pocket” (PiP) strategy for certain patients with infrequent, repeated HIV exposures who decline to use PrEP.

Certain promising strategies are available to overcome these barriers. Similar to HIV PrEP, all adolescents and adults should be informed about nPEP as an option for HIV prevention if a potential exposure has occurred (*4*). Enhanced education for health care professionals along with additional supports, such as tailored clinical guidelines, checklists, order sets, and other clinical support tools, might increase guideline-consistent nPEP clinical care (*107*,*109*,*122*,*123*). In addition, multidisciplinary and integrated service programs that include pharmacists, sexual assault forensic examiners, patient navigators, mental health care professionals, and community partnerships with pharmacies or syringe service programs might improve nPEP initiation, continuity of treatment, completion, and follow-up care (*106*,*122*–*127*). If a patient has difficulty accessing medications, a social worker, case manager, patient navigator, or other support staff member should be engaged to assist with medication access.

Systems-level strategies are needed to help ensure access to nPEP. Local health care professionals, health systems, and jurisdictions should identify barriers to nPEP care and create protocols to ensure timely and equitable nPEP access for all populations.

For certain persons seeking services for nPEP, a “PEP-in-pocket” (PiP) approach might be useful. The PiP approach provides education and a 28-day supply of recommended HIV PEP medication regimen to persons with low-frequency, high-risk HIV exposures who decline to use one of the available PrEP regimens. Persons are educated to immediately start their PiP supply if they have a possible HIV exposure. Persons who initiate PiP are followed up in clinic as soon as possible for further evaluation, including for HIV and other STI testing, and assessment of whether PEP should be continued. Centers using this approach have described substantial success, including a high level of correct use and follow-up, and no reported HIV acquisitions to date (*127*–*131*). More comprehensive data including use of this approach in diverse populations and settings will be useful to guide future recommendations.

## Discussion and Conclusion

These nPEP recommendations and clinical considerations provide a safe and effective strategy to prevent HIV infection. The continued occurrence of tens of thousands of HIV diagnoses annually in the United States indicates the importance of implementing the full spectrum of HIV prevention options, including nPEP (*1*). Increasing HIV nPEP knowledge and awareness, supporting nPEP adherence, and improving nPEP access are all core strategies available to enhance HIV prevention efforts in the United States (*4*).

Multiple core components of U.S. nPEP recommendations were originally based on data from animal models and small observational studies (*4*). Evidence accumulated since then suggests that nPEP, when used as currently recommended, including a 72-hour initiation window, a 28-day duration, and a newer generation 3-drug ART regimen, is safe, generally well tolerated, and likely to reduce the risk for HIV acquisition (*4*,*46*,*132*).

Important questions remain about optimal HIV nPEP delivery. New empirical data in the following topic areas might contribute to updated HIV nPEP practices in the future:

- **nPEP time to initiation.** The recommendation to offer HIV nPEP up to 72 hours after a known or potential HIV exposure was based on animal models with known limitations, including differences in host and viral biology, and different ARVs used as PEP than the currently available regimens (*25*,*26*). To date, no human data are available to define a different window of time for nPEP initiation. CDC’s oPEP guidelines include discussion of HIV PEP use >72 hours from exposures with substantial likelihood of HIV transmission (*4*). More information is needed to determine whether the time to nPEP initiation window can be shortened or lengthened. Although the efficacy end point of the nPEP initiation time window might be undefined, broad consensus and compelling human data support the recommendation to initiate nPEP as soon as possible, preferably within 24 hours of HIV exposure (*24*).
- **nPEP duration.** The recommendation to prescribe HIV nPEP for 28 days was based on animal data with known limitations (*29*). The updated recommendation maintains the effective 28-day current standard of care while acknowledging medication dispensing practices that might make it more practical to prescribe a 30-day rather than a 28-day course. nPEP users might be instructed that the course is complete after 28 days. No data are currently available to define an alternative nPEP duration; however, newer animal model data suggest a shorter nPEP course might be effective, especially if initiated <24 hours from exposure (*37*,*40*). More information is needed to define the nPEP duration that provides prevention efficacy similar to the current standard of care when implemented in real-world settings where the time between HIV exposure and nPEP initiation varies.
- **2-drug nPEP regimens.** The recommendation to provide a 3-drug nPEP regimen was based on nPEP animal model data and HIV treatment data from clinical trials (*30*,*47*). The current recommended 3-drug nPEP regimens are generally well tolerated and expected to provide robust suppression of viral replication and protection against drug resistance (*4*,*20*,*46*,*47*,*132*). An Australian study comparing nPEP regimens of two NRTIs to regimens of two NRTIs plus lopinavir/ritonavir (LPV/r) found that 2-drug regimens were not associated with increased risk for HIV acquisition compared with 3-drug regimens among GBMSM (*133*). Certain experts have suggested that using a 2-NRTI combination could increase nPEP accessibility and reduce time to nPEP initiation because of the widespread availability of certain such combinations (*133*). More studies are needed evaluating 2-drug regimens and their efficacy as nPEP among diverse populations (e.g., women) and settings (*4*,*20*,*46*,*47*,*132*).
- **Long-acting injectable ART for nPEP.** Injectable cabotegravir is a recommended PrEP option, and injectable cabotegravir plus rilpivirine is a treatment option for persons with HIV who have achieved viral suppression on a recommended initial regimen (*47*,*134*). However, available data are not able to guide recommendations about use of injectable or other long-acting agents for nPEP (*44*,*133*). Although long-acting agents offer the potential for a convenient, simple nPEP regimen, unanswered questions include prevention efficacy and the risk for acquiring INSTI-resistant HIV.
- **PiP.** Certain centers have had success with offering a supply of nPEP medications (PiP) to selected persons with low-frequency, high-risk HIV exposures who decline to use PrEP (*127*–*129*). Persons who initiate nPEP using their PiP supply should follow up for clinical evaluation as soon as possible. This approach has the potential to substantially reduce time to nPEP initiation, but questions remain about cost implications as well as implementation considerations across diverse populations and settings in the United States.

## Future Research

A randomized controlled trial has never been conducted to assess the efficacy of ARV regimens used for nPEP, the frequency of HIV acquisition based on time from HIV exposure to nPEP initiation, or the length of an nPEP course. Instead, recommendations for nPEP have been based on observational studies in humans and studies in non-human primates. In addition, information about the efficacy, safety, and pharmacokinetic properties of ARV medications used to treat HIV have guided selection of nPEP regimens. Although randomized controlled trials provide an ideal study design to understand optimal properties of an nPEP regimen, the ethics are problematic for a placebo-controlled trial or regimens shorter than the effective 28-day course (*4*). A large number of participants would need to be enrolled for sufficient statistical power to assess these outcomes, making such studies logistically difficult. Data from large health care databases and meta-analyses might provide insight about whether shorter durations of nPEP might be sufficient for protection. Such analyses also can potentially provide information about the safety and ethics of a PEP clinical trial.

Observational, pharmacokinetic, and animal studies are needed to understand the safety and effectiveness of novel nPEP regimens that have potential to increase adherence with and completion of an nPEP course. A potential example is a one-time injection of a long-acting ARV medication combined with oral loading dosing. Safety and effectiveness studies also are needed for other newer ARV regimens, including regimens containing fewer than three agents. Studies of the performance of testing strategies with HIV Ag/Ab, RNA, and rapid (point-of-care) HIV tests and optimal post-nPEP testing intervals can guide recommendations for testing at nPEP initiation and completion.

nPEP is a key component of PrEP care as an intervention for users of on-demand or daily oral PrEP who might have adherence challenges. Hybrid implementation and effectiveness studies of PiP models for PrEP users are needed. Although PiP has been found effective in a Canadian study (*130*), understanding its use in U.S. populations of men who have sex with men is needed. Studies also are needed to identify optimal models and practices to transition an nPEP user to PrEP. Communication and education strategies are needed to increase both provider and community awareness of nPEP. Finally, to assess public health activities that aim to increase nPEP use by persons who might benefit from it, surveillance measures are needed for monitoring (*4*,*135*).

## Footnotes

* These authors contributed equally to this work.

## Appendix A Known or Possible HIV Exposure Scenarios and Associated Nonoccupational Postexposure Prophylaxis Considerations

**Table:**

Exposure	nPEP considerations	Rationale and references
**All known or possible exposures**	The NCCC PEPline is available for clinical consultation on nPEP questions, including questions about HIV exposure and nPEP indications, at 888-448-4911 or https://nccc.ucsf.edu/clinician-consultation/pep-post-exposure-prophylaxis	Persons with a recent HIV exposure might be concerned about disclosing all details of the behaviors that might have exposed them to HIV. Multiple factors might contribute to this reluctance, including trauma from sexual assault, concerns about stigma, and medical mistrust, among others (*1**–**4*). Health care professionals can prescribe nPEP to persons who request it, even if they are unable to elicit all details necessary to assess HIV acquisition risk.
**Sexual exposure**		
Sexual exposure with an intact condom	nPEP not recommended	When used as recommended, condoms are highly effective at preventing HIV transmission (*5**–**7*). If the condom was not used consistently or correctly throughout the sexual encounter to prevent body fluid exposure, consider the situation as though the sexual exposure was without a condom.
Sexual exposure in which the exposed person is taking HIV PrEP as recommended	nPEP not recommended	PrEP taken as directed is highly effective at preventing HIV acquisition, and nPEP is not generally recommended for persons who are consistently taking PrEP (*8**–**11*). However, nPEP may be considered in certain scenarios, including 1) persons who have recently started PrEP and might not have yet reached maximum protection (*12*), 2) persons who have missed PrEP doses, 3) persons using an intermittent regimen outside of current CDC PrEP guideline recommendations (*12*), and 4) persons with exposure to a source without sustained viral suppression and resistance to PrEP components.
Sexual exposure in which the source has sustained HIV viral suppression defined for the purposes of nPEP as HIV treatment >6 mos, consistent high level of ARV adherence, and HIV RNA <200 copies/mL or undetectable on all laboratory assessments in the last year, with the most recent HIV RNA test result within 1–2 mos of exposure	nPEP not routinely recommended	For sources with sustained HIV viral suppression, the available data indicate that they will not transmit HIV sexually (*13**–**16*). It is important that health care professionals establish that the source has continued to have a high level of ARV adherence in the time since the last HIV RNA test. Individual situations for persons seeking nPEP might differ from the exposure scenarios in the studies informing this recommendation. Consultation with nPEP experts (e.g., NCCC) might be useful when individual questions arise.
Anal or vaginal intercourse without a condom when the source has HIV with detectable viremia or unknown viral suppression status	nPEP recommended	The recommendation to routinely offer nPEP in this scenario is based on the potential for HIV acquisition (Table 2) and the overall safety and tolerability of current nPEP regimens (*17*).
Anal or vaginal intercourse without a condom when it is not known whether the source has HIV	Case-by-case determination	Quantifying the likelihood of HIV acquisition in this scenario is not possible. The decision to initiate nPEP must be based on individual risk assessment and shared decision-making. Health care professionals can use available resources to consider how sexual behaviors (Table 2) and other factors (e.g., population HIV prevalence) influence the overall likelihood of HIV acquisition (*18*). Other factors that might influence the likelihood of HIV transmission, including trauma and concurrent STIs, should also be considered (*17**,**19**,**20**,**21*). The high level of safety and tolerability of newer nPEP regimens also might factor into discussions when the risk for HIV acquisition is unknown (*17*). HIV nPEP should be offered to survivors of sexual assault as part of comprehensive post-assault services when the assault included any contact associated with substantial risk for HIV transmission and the source’s HIV status is unknown (*21*).
Oral-genital sexual contact without a condom, regardless of source’s HIV status	nPEP not routinely recommended	The likelihood of HIV transmission with oral-genital sexual contact is low (*22*). On the basis of the individual risk assessment, health care professionals might choose to offer nPEP in the presence of other factors that might increase the risk for HIV transmission (e.g., trauma with blood exposure, non-intact mucus membranes, or high-level viremia) (*23*).
**Injection drug exposure**		
Sharing needles or other drug injection equipment that resulted in exposure to the source’s blood when the source has sustained HIV viral suppression (HIV treatment >6 mos, consistent high level of ARV adherence, and HIV RNA <200 copies/mL or undetectable on all laboratory assessments in the last year, with the most recent HIV RNA test result within 1–2 mos of exposure)	Case-by-case determination	Extrapolation from studies examining HIV transmission risk from sexual exposure or accidental percutaneous exposure to persons with viral suppression suggests that HIV transmission is not expected in this setting (*14**–**16**,**24**,**25*). However, data directly addressing this exposure scenario are lacking, and individual situations for persons seeking nPEP services might differ from available studies. Health care professionals should establish that the source has continued to have a high level of ARV adherence in the time since the last HIV RNA test before deciding that the source meets criteria for sustained viral suppression. nPEP decisions must be based on an individual risk assessment and shared decision-making.
Sharing needles or other drug injection equipment that resulted in exposure to the source’s blood, when the source has HIV with detectable viremia or unknown viral suppression status	nPEP recommended	The scenario represents a substantial risk for HIV acquisition and nPEP is recommended (*26*).
Sharing needles or other drug injection equipment that resulted in exposure to the source’s blood, when it is not known whether the source has HIV	Case-by-case determination	Quantifying the risk for HIV acquisition is not possible when the HIV status of injection partners is unknown. Sharing needles and other drug injection equipment that results in blood exposure is associated with increased risk for HIV transmission compared with injection drug use without sharing (*26**,**27*). The high level of safety and tolerability of newer nPEP regimens also might factor into discussions when the risk for HIV acquisition is unknown (*17*).
**Other exposures**		
Human bites	nPEP not routinely recommended	HIV transmission through human bites is rare but has been reported (*28**–**30*). Human bites with no blood exposure are not an indication for nPEP. Exposure to visibly bloody saliva presents some risk for HIV acquisition, especially if the source is known or suspected to have detectable HIV viremia; in such cases nPEP should be considered (*31*). Clinical evaluation of human bites should include the possibility that both the bitten person and the person biting might have been exposed to HIV and other bloodborne pathogens such as HCV.
Oral-oral exposure (kissing), mutual masturbation, any exposure to body fluids not associated with HIV transmission (e.g., tears, sweat, urine, nasal secretions, and saliva)	nPEP not routinely recommended	Other than exceptional circumstances where blood contamination is visible and the exposed person’s mucus membranes or non-intact skin were affected, these exposures represent negligible risk for HIV transmission, and nPEP is not indicated (*23*).
Blood or other infectious body fluid splash to non-intact skin or mucous membranes	Case-by-case determination	Rare cases of HIV acquisition from mucocutaneous exposure to splashed infectious body fluids have been described (*32**,**33*). Mucus membrane or non-intact skin exposure to blood or infectious body fluid (e.g., semen, cervicovaginal secretions, breast milk, or any visibly bloody secretions) from a source with known detectable HIV viremia is an indication for nPEP, similar to oPEP recommendations (*34*). Splashes from body fluids not associated with HIV transmission (e.g., tears, sweat, urine, nasal secretions, and saliva) when not visibly bloody are not an indication for nPEP.
Injury from a discarded needle in the community	nPEP not recommended unless exceptional circumstances	nPEP is not typically indicated for accidental injuries from discarded needles in community settings (*35*). No confirmed reports are available of HIV acquisition from this route of exposure. Viral characteristics, including rapid decline in HIV infectivity outside of the human body, likely contribute to the reduced risk for transmission via discarded needles compared with percutaneous injury in health care settings (*36**,**37*). Individual risk assessments are required. In exceptional circumstances, certain experts would offer nPEP (e.g., penetrating injury from a freshly bloody discarded needle used by a person who injects drugs).

**Abbreviations:** ARV = antiretroviral; HCV = hepatitis C virus; NCCC = National Clinician Consultation Center; nPEP = nonoccupational postexposure prophylaxis; oPEP = occupational postexposure prophylaxis; PrEP = pre-exposure prophylaxis; STI = sexually transmitted infection.

## Appendix B A Summary Discussion of Characteristics of Antiretroviral Agents Used for Nonoccupational Postexposure Prophylaxis

### nPEP Efficacy for HIV Prevention

No randomized, placebo-controlled clinical trial evaluating the efficacy of nonoccupational postexposure prophylaxis (nPEP) for HIV prevention has been performed. However, data relevant to nPEP guidelines are available from multiple prospective, open-label randomized and nonrandomized experimental studies, longitudinal and observational cohort studies, and case studies of nPEP use. The 2025 nPEP guideline update adds evidence from January 2015 to January 2024. A systematic literature review was conducted according to Preferred Reporting Items for Systematic reviews and Meta-Analyses (PRISMA) reporting guidelines. Literature searches were performed in Medline, Embase, PsycINFO, Cochrane Library, CINAHL, and Scopus databases. Search terms included “HIV post exposure prophylaxis,” “post-exposure prophylaxis,” “nPEP,” “nonoccupational postexposure or post-exposure prophylaxis,” “HIV postexposure or post-exposure prophylaxis,” “post exposure or post-exposure prevention,” “non-occupational,” “non-PEP,” “NOPEP,” “PEP,” “post-exposure prophylaxis after sexual exposure,” and “PEPSE.” Duplicates were identified and removed using the Endnote automated “find duplicates” function with preferences set to match on title, author, and year. Additional deduplication occurred during the review and categorization process. Studies included in this literature review were published in peer-reviewed journals or in CDC’s *Morbidity and Mortality Weekly Report.* The nPEP guideline update team defined key outcomes relevant to the nPEP topic areas. Exclusion criteria included no full-text available; non-human study; not relevant to HIV PEP; publication withdrawn or otherwise inaccessible; commentary or otherwise non–peer-reviewed studies except relevant conference abstracts; study protocol with no data; studies outside of the United States; nPEP epidemiology before 2018 (e.g., nPEP awareness and use); only about oPEP unless specifically on medications, regimens, adherence, outcomes, or side effects; and prevention of perinatal or mother-to-child transmission only. New evidence-based recommendations were developed using the Grading of Recommendations, Assessment, Development, and Evaluation (GRADE) framework.

Alongside the systematic literature review, the nPEP work group also considered evidence from clinical trials and observational studies that used antiretroviral therapy (ART) for the purpose of HIV treatment to enhance understanding of the HIV current standards of care, which are not reflected in the GRADE tables but are discussed here. These ART agents include the nucleoside and nucleotide reverse-transcriptase inhibitors (NRTIs), nonnucleoside reverse-transcriptase inhibitors (NNRTIs), protease inhibitors (PIs), integrase strand transfer inhibitors (INSTIs), a fusion inhibitor (FI), chemokine (C-C motif) receptor 5 (CCR5) antagonists (entry inhibitors), and postattachment inhibitors. Only ART agents approved by the Food and Drug Administration (FDA) for treatment of HIV infection were reviewed or included in these guidelines, although none of these agents has an FDA-approved indication for administration as nPEP. Newer data discussed in this report continue to support the assertion that nPEP initiated soon after exposure and continued for 28 days with sufficient medication adherence can reduce the risk for acquiring HIV infection after nonoccupational exposures. In many studies, HIV seroconversions have most commonly been attributed to ongoing risk behavior after completion of the nPEP course (*1*–*9*). However, certain studies have suggested that delayed initiation of nPEP, low adherence, and early primary HIV infection at the time of nPEP initiation as risks for HIV acquisition (*1*,*3*,*10*).

### nPEP Studies by Regimen

**INSTI-Based Regimens**

*Bictegravir (BIC).* Two prospective, nonrandomized open-label trials have evaluated BIC, emtricitabine (FTC), and tenofovir alafenamide (TAF) as once daily nPEP for 28 days (n = 164) (*11*,*12*). Both studies noted high completion rates (90% and 96%) by self-report with zero seroconversions (*11*,*12*). When comparing regimen completion rates to PEP regimens from earlier clinical trials, including 1) zidovudine (AZT)/lamivudine (3TC) plus a PI, 2) twice daily raltegravir (RAL) plus tenofovir disoproxil fumarate (TDF)/FTC, and 3) co-formulated elvitegravir (EVG)/cobicistat (c)/TDF/FTC, BIC/FTC/TAF, had significantly higher completion rates than the other regimens (38.8%, 57.0%, and 71.0%, respectively [p<0.05]) (*11*). In addition, BIC/FTC/TAF was less likely to be associated with side effects than historical studies, including RAL- and EVG-based regimens (*11*).

*Dolutegravir (DTG).* Three studies (two cohort and one open-label single arm trial) have evaluated DTG-based regimens, including in combination with TDF/FTC and abacavir (ABC)/3TC as PEP (n = 1,134) (*13*–*15*). No studies compared BIC with DTG-based regimens. Studies containing DTG noted zero HIV seroconversions. Among studies reporting individual completion rates for DTG-based regimens, completion rates ranged from 64% to 94% (*13*–*15*). Rates of self-reported adherence to PEP were up to 98% (*13*–*15*). Premature PEP cessation due to adverse events was rare (*13*–*15*).

*Elvitegravir (EVG).* Six studies have examined regimens containing EVG as PEP (n = 2,351) (*2*,*6*,*9*,*13*,*16*–*18*). In three studies, no seroconversions were noted among participants who were administered EVG-based regimens (*9*,*13*,*16*). In a fourth study, one seroconversion occurred in the EVG/cobicistat (c)/TDF/3TC arm at day 90 in a person with multiple high-risk exposures to HIV before and after starting PEP (*6*). One study did not report HIV follow-up testing and one study reported a person who acquired HIV as a possible nPEP failure, but the nPEP regimen was not specified (*9*,*18*). Completion rates for EVG-based regimens ranged from 44% to 92% (*6*,*9*,*13*,*16*–*18*). In studies with comparison with other regimens, completion and adherence rates were higher for EVG-based regimens (including with TAF) than for those consisting of NNRTI plus two NRTIs (89% [CI = 88%–90%; n= 2,786]) or PI plus two NRTIs (80% [CI = 79%–81%; n = 12,903]) (*6*,*13*). Mixed evidence exists comparing EVG with other INSTIs for PEP. However, a separate study suggested RAL/TDF/FTC has higher rates of completion up to 96% (CI = 94%–98% [n = 866]), with EVG-based regimens being similar to DTG plus TDF/FTC (87% [CI = 84%–90%; n = 704]) (*13*,*16*). Most adverse events were mild and did not result in nPEP discontinuation (*6*,*13*,*16*). Adverse events were less frequent than in comparator groups (e.g., lopinavir and ritonavir [LPV/r]) (*6*,*9*,*13*,*16*–*18*).

*Raltegravir (RAL).* Fourteen studies examined adherence, tolerability, or efficacy of RAL-based regimens for PEP (n = 3,101) (*3*–*5*,*7*,*13*,*19*–*27*). Multiple studies reported HIV seroconversions. One seroconversion occurred in a patient reported to be on RAL at day 90 with known multiple potential sexual risk exposures before and after receiving nPEP (*4*). Three studies reported at least one seroconversion; however, the PEP regimen was not specified, and all but one seroconversion across all these studies occurred in persons who had continued subsequent high-risk behaviors (*5*–*7*). Another study had three HIV acquisitions in persons receiving RAL-based PEP but was not significantly different from compared regimens in multivariate regression (*3*). Six studies reported no seroconversions (*7*), and three studies did not report the number of seroconversions (*3*,*4*,*20*–*27*).

The completion rates of RAL-based regimens ranged from 32% to 96% (*3*–*5*,*7*,*13*,*19*–*27*). In studies comparing the completion rates of multiple regimens, RAL-based regimens had higher rates than older regimens (e.g., LPV/r) (*4*). However, commonly reported issues with adherence that required a regimen modification included failure to consistently take the second daily dose of RAL (*19*). Multiple studies reported discontinuations due to adverse events, but these events occurred less frequently than with older recommended regimens, suggesting better tolerability of RAL (*4*,*21*,*23*).

**Long-Acting Injectables**

Cabotegravir (CAB), a newer INSTI, is available co-packaged with rilpivirine (RPV), or separately for use as HIV pre-exposure prophylaxis (PrEP). CAB-RPV is available as extended-release injectable suspension administered intramuscularly (IM) as HIV treatment once every 4–8 weeks. The literature review did not reveal any studies examining the prescription of IM-administered long-acting cabotegravir-based ART regimens (i.e., CAB-RPV) as an alternative PEP regimen among any population, including in health care personnel with an occupational exposure to HIV or with nonoccupational exposures to HIV. A recent animal study indicated that PEP with long-acting CAB-RPV was partially effective and demonstrated late breakthrough infections, highlighting the limitations of this regimen for PEP (*27*). Due to the lack of data on safety, tolerability, and efficacy in a PEP setting, CAB-RPV was not included in the list of preferred or alternative regimens in this guideline update.

**PI-Based Regimens**

Twelve reports examined adherence, tolerability, or efficacy of PI-based regimens, including lopinavir (LPV)- or darunavir (DRV)-based regimens (n = 14,398) (*10*,*13*,*20*,*28*–*40*). In studies reporting seroconversions, 18 occurred; at least nine were considered nPEP failures (*10*,*13*,*30*,*32*). No reports of HIV acquisition were among persons completing LPV-based PEP; two HIV acquisitions were among persons who stopped LPV-based PEP early during the course. PI-based regimens have among the lowest completion rates, ranging from 42% to 80% (*10*,*13*,*20*,*28*–*40*). In studies that compared different regimens, PI plus two NRTIs had lower completion rates than INSTI- and NNRTI-based regimens, with atazanavir and ritonavir (ATV/r) and nelfinavir (NFV) among the lowest (*13*). Adverse events were significantly higher with PI-based regimens than with INSTI-based regimens (p<0.05) (*20*). Early discontinuation is not uncommon (*13*,*20*,*28*–*32*). Case reports exist of LPV/r discontinuation due to serious adverse events (suspected acute interstitial nephritis and drug interaction with dihydroergotamine) (*35*,*37*,*40*). An observational study of DRV/r plus TDF/FTC with self-reported questionnaires (n = 51) demonstrated a discontinuation rate of 47% with six cases of discontinuation (12%) being treatment related (*33*). PI-based regimens containing AZT have an increased risk for drug discontinuation (relative risk = 9.33 [95% CI = 1.34–65.23]) due to adverse effects of medication related to gastrointestinal complications (*29*).

A 2015 systematic review of nPEP regimens that included 25 studies found that of 10 studies with 1,755 initiations, nPEP completion rates were highest for DRV/r plus TDF/FTC (94% [CI = 90%–98%]), then LPV/r plus TDF/FTC (71% [CI = 44%–97%]), and lowest for LPV/r plus AZT/3TC (59%) (*8*). The discontinuations due to drug reactions were highest for ATV/r plus AZT/3TC (21%). nPEP failure as determined by HIV seroconversion was rare and could not be compared across regimens because of the paucity of events and different protocols for longer-term monitoring after nPEP provision (*8*).

**NNRTI-Based Regimens**

Seven studies examined adherence, tolerability, or efficacy of NNRTI-based regimens as PEP (n = 3,580) (*13*,*41*–*46*). Overall, NNRTI-based regimens containing RPV had high completion rates, ranging from 81% to 92% (95% CI = 85%–96%) (*13*,*42*,*45*). A multicenter, open-label, nonrandomized trial of 100 men who have sex with men who received RPV/TDF/FTC once daily for 28 days demonstrated overall high adherence of >90% by self-report in 90% of persons despite frequent experiences of one or more clinical adverse events (88%) (*42*). Overall, few premature discontinuations of RPV due to adverse events were observed, mainly due to gastrointestinal intolerance (*41*,*45*,*46*). NNRTI regimens containing efavirenz (EFV) had lower completion rates (69%–71%) with lower tolerability (*43*,*44*). One cohort study found EFV as a significant factor associated with PEP noncompletion (OR = 37.8 [CI = 4.2–342.3; p<0.01]), with at least 10 persons discontinuing prematurely due to severe dizziness (*43*). Despite this, in the seven studies examining NNRTI-based regimens, zero seroconversions occurred (*13*,*41*–*46*).

### Potential Risks Associated with nPEP

Concerns regarding potential risks associated with nPEP as a clinical HIV prevention intervention include the occurrence of serious adverse effects from the short-term use of ART medications by otherwise healthy persons without HIV infection. Another concern is potential selection for drug-resistant strains of virus among those who acquire HIV despite nPEP use (particularly if medication adherence is inconsistent during the 28-day course or if the source transmits resistant virus).

**ART Side Effects and Toxicity**

*INSTI-Based Regimens.* All INSTIs are typically well tolerated (*47*). Insomnia, depression, and suicidal ideation, primarily in patients with a history of psychiatric illnesses, have been reported rarely in patients receiving INSTI-based regimens (*47*). In addition, initiation of INSTI-based regimens has been associated with greater weight gain than with NNRTI- or PI-based regimens, although this generally is observed with longer therapy than required for nPEP.

Fifteen studies have reported on the tolerability, side effects, and toxicity of INSTI-based regimens for use as nPEP. Reported side effects of BIC and DTG were mostly mild or self-limiting and did not result in nPEP discontinuation (*11*–*13*,*48*). The most common side effects of BIC- and DTG-based regimens include fatigue, headache, nausea or vomiting, and diarrhea. The most commonly reported adverse reactions of moderate-to-severe intensity to DTG-based regimens were insomnia and headache. Adverse events to DTG resulting in study drug discontinuation were rare but have been reported (headache [1%]) (*14*). Abnormal laboratory values, including elevated creatinine and liver transaminases, were observed rarely for BIC- and DTG-based regimens and resolved after PEP regimen completion (*11*,*12*,*48*). In an open-label, nonrandomized phase IV trial of 52 persons, BIC/FTC/TAF was less likely to be associated with any symptom compared with historical regimens containing AZT/3TC/PI (*11*). BIC/FTC/TAF also was less likely to be associated with diarrhea, loose stools, or headache than AZT/3TC/PI, RAL/FTC/TDF, and EVG/c/FTC/TDF; less likely to be associated with fatigue than EVG/c; and less likely to be associated with dizziness than RAL (*11*). Nonrandomized trials of EVG/c reported higher frequency of mild adverse events with nPEP use than observed in persons with HIV infection, most commonly abdominal discomfort, bloating, diarrhea, fatigue, nausea, vomiting, headache, or dizziness (*6*,*16*,*49*). These adverse events occur less frequently than with PI-based regimens (e.g., LPV/r) (*6*,*9*,*13*,*16*–*18*).

Although rare, side effects can occur with RAL for nPEP and include gastrointestinal discomfort, nausea, dizziness, and headache (*3*,*4*,*21*–*25*,*50*). Rare cases of skeletal muscle toxicity, thrombocytopenia, and severe systemic cutaneous reactions resembling Stevens-Johnson syndrome also have been reported (*51*–*54*). Side effects of RAL occur less frequently compared with LPV/r or ATV (*21*,*22*,*24*,*25*). Abnormal laboratory values observed include elevated creatinine and liver transaminases (*23*).

Extrapolating data from HIV treatment trials, multiple comparisons of boosted PI- and INSTI-based regimens used as HIV treatment revealed that the INSTI-based regimens were better tolerated and resulted in fewer treatment discontinuations (*47*,*55*–*57*). Compared with boosted PI-based regimens and NNRTI-based regimens, INSTI-based regimens were more likely to have viral suppression and are among the most effective agents in suppressing HIV viral load (*55*,*58*–*61*).

Among the INSTI-based regimens, BIC- and DTG-based regimens have a higher barrier to resistance, lower pill burden, and higher completion rates than the first-generation INSTI-based regimens that contain EVG or RAL (*47*,*62*,*63*). EVG-based regimens also contain cobicistat, a strong cytochrome P450 inhibitor, which increases the potential risk for drug–drug interactions (*47*).

Transmitted and treatment-emergent resistance has been reported rarely with DTG- or BIC-based treatment regimens (*64*–*67*). Data from two randomized trials demonstrated that, in terms of virologic efficacy, DTG plus 3TC was noninferior to a 3-drug regimen of DTG plus TDF/FTC (*68*,*69*). No treatment-emergent resistance was observed in either the 2-drug or the 3-drug group (*68*,*69*). HIV treatment clinical trials have demonstrated BIC-based regimens to be noninferior to DTG-based regimens (*70*,*71*).

**PI-Based Regimens**

Multiple side effects appear to be class-specific, whereas others are agent-specific. PI class-specific side effects include metabolic complications (e.g., insulin resistance, diabetes, dyslipidemia, and lipodystrophy) and other adverse reactions (e.g., hepatotoxicity) (*72*–*75*). However, the propensity to cause side effects differs by PI and pharmacokinetic (PK)-enhancing agent. Most drug–drug interactions with PIs arise from the pharmacological boosting agents, ritonavir and cobicistat.

Five studies examined adherence and tolerability of PI-based nPEP regimens (*20*,*28*,*29*,*32*,*34*,*76*). Multiple observational and randomized, noninferiority studies have suggested improved tolerability, lower adverse events overall, and fewer moderate-to-severe events for boosted DRV than boosted LPV and ATV, due in part to hyperbilirubinemia associated with ATV (*28*). Commonly reported side effects of DRV/r include fatigue, nausea, diarrhea, loss of appetite, and headache; studies comparing DRV/r with INSTIs revealed significantly less side effects for INSTIs (p<0.0001) (*20*,*28*,*76*). Other reactions observed with DRV/r include skin rash, which is usually mild-to-moderate in severity and self-limited, and elevated liver transaminases. Case reports of TDF-induced Fanconi’s syndrome are described in the nPEP literature, one in combination with DRV/r (*39*). ATV and cobicistat (ATV/c) and ATV/r can cause fatigue and gastrointestinal side effects, including diarrhea (*32*). Nephrolithiasis, nephrotoxicity, and cholelithiasis have been reported for ATV used in treatment regimens (*77*–*81*). Adverse events also have been reported with LPV/r for nPEP, including nausea, vomiting, diarrhea, fatigue, headache, acute kidney injury, and ergotism with acute limb ischemia (*34*–*37*).

In studies for the treatment of HIV infection, large observational cohorts demonstrated an association between certain PIs (DRV/r, LPV/r) and an increased risk for cardiovascular events; however, this association was not observed with ATV (*82*–*85*). Further study is needed to determine the clinical significance of this finding for nPEP with a 28-day course. Boosted ATV, like boosted DRV, has relatively few metabolic adverse effects compared with older boosted-PI regimens; however, ATV/r had a higher rate of adverse effect–associated drug discontinuation rate than DRV/r and RAL in a randomized clinical trial (*55*). Certain studies have demonstrated that unsuspected drug–drug interactions were more common among persons with HIV infection taking PIs compared with NNRTIs or NRTIs (*86*).

Boosted-PI (PK-enhanced) regimens have greater potential for drug–drug interactions than other regimens. However, nPEP observational studies and treatment clinical trials have suggested boosted PI-based regimens (boosted DRV) have excellent completion rates and tolerability with low rates of transmitted and treatment-emergent resistance (*87*). DRV/c/TAF/FTC is available as a fixed-dose single tablet once-per-day regimen that can be considered in certain clinical situations.

**NNRTI-Based Regimens**

Although no explicit class-specific adverse events have been reported, two commonly used NNRTIs (EFV and RPV) can result in QTc prolongation, skin rash, and neurologic and psychiatric side effects, including depression. Seven studies examined adherence and tolerability of NNRTI-based regimens as nPEP (*13*,*41*–*46*). Multiple multicenter, nonrandomized studies of RPV/FTC/TDF reported high proportions (up to 88% of participants) of one or more clinical adverse events, including fatigue, dizziness, nausea, gastrointestinal intolerance, headache, and sleep disorders (*41*,*42*,*45*). Although most adverse events were mild and self-limited, few premature discontinuations occurred (*41*,*42*,*45*). Laboratory abnormalities included elevated creatinine (*41*,*42*). Doravirine (DOR) and RPV are generally better tolerated than EFV (*88*). EFV-based PEP regimens are associated with premature discontinuation, mostly due to severe dizziness (*43*). Side effects observed with EFV include central nervous system (CNS) toxicity, elevated hepatic transaminases (including fulminant hepatitis), and QT interval prolongation (*43*,*89*–*92*). Thus, EFV is avoided in persons with severe liver disease (Child-Pugh classes B and C) and psychiatric illness. Other side effects include rash and hyperlipidemia (*43*).

The major disadvantages of currently available NNRTIs are the prevalence of NNRTI-resistant viral strains in ART-naive patients and the drugs’ low barrier for the development of resistance. The first-generation NNRTIs (e.g., EFV and NVP) require only a single mutation to confer drug resistance (*47*). Despite this, EFV-based regimens have excellent virologic efficacy, although the relatively high rate of adverse events (e.g., CNS related) can lead to nPEP discontinuation (*93*,*94*). Two controlled trials compared RPV with EFV in treatment-naïve patients in combination with two NRTIs and demonstrated comparable proportions of viral suppression at 48 weeks with improved tolerability of RPV. Compared with EFV and DRV/r, DOR is noninferior with excellent virologic efficacy and has fewer metabolic adverse effects and less potential for drug–drug interactions (*88*,*94*,*95*).

**NRTI-Containing Regimens**

Although less common with newer NRTIs, a hallmark toxicity of the NRTI class is mitochondrial toxicity. This toxicity might manifest as peripheral neuropathy, myopathy, pancreatitis, lipoatrophy, hepatic steatosis, and lactic acidosis (*96*–*100*).

Five studies had TAF-containing regimens as nPEP (n = 479) (*2*,*11*–*13*,*46*). Among four studies reporting completion rates of various TAF-containing regimens, the completion rates ranged from 82% to 96% without any HIV seroconversions (*2*,*11*–*13*,*46*).

Tenofovir can lead to kidney and bone toxicities, especially when used with a PK booster; however, TAF is associated with less renal and bone toxicity compared with TDF because it achieves lower plasma tenofovir concentrations (*101*,*102*). TAF might be associated with higher blood lipid levels than TDF (*103*). Both FTC and 3TC have been well tolerated with no significant treatment-limiting adverse effects (*104*). Rarely, 3TC has been associated with pancreatitis; this risk might be higher in children (*105*,*106*).

Less commonly used NRTIs include ABC and AZT. ABC is generally avoided due to concerns for developing an ABC hypersensitivity reaction and the need for HLA-B5701 testing. In addition, ABC generally is avoided in persons with coronary artery disease due to possible risk for myocardial infarction (*107*,*108*). Adverse reactions reported with AZT include headache, malaise, anorexia, nausea, vomiting, lactic acidosis, and loss of limb fat. A study examining nPEP longitudinal trends suggested that TDF-containing regimens were associated with significantly higher completion rates than AZT-containing regimens (adjusted OR = 2.80 [95% CI = 1.69–4.63; p<0.001]) (*106*).

### PK Boosters

PK enhancement, or PK boosting, for ART regimens contains either cobicistat or ritonavir. Regimens with cobicistat and ritonavir, both potent CYP3A enzyme inhibitors, might lead to significant interactions with medications metabolized by this enzyme (*47*). Cobicistat also inhibits active tubular secretion of creatinine, resulting in increases in serum creatinine and reduction in estimated creatinine clearance, without reducing glomerular function (*109*,*110*). Adverse effects of ritonavir include gastrointestinal intolerance, hyperlipidemia, bitter aftertaste, and malaise, some of which are dose-related (*47*).
