# *Erratum:* Vol. 74, No. RR-1

**DOI:** 10.15585/mmwr.mm7435a3

**Published:** 2025-09-18

In the report “Antiretroviral Postexposure Prophylaxis After Sexual, Injection Drug Use, or Other Nonoccupational Exposure to HIV — CDC Recommendations, United States, 2025,” the following errors occurred:

On page 6, in the second column of Box 2, the third bullet should have read, “For persons with long-acting injectable PrEP ARV exposure during the past **6 months,** a diagnostic HIV nucleic acid test (NAT) is recommended at the initial medical evaluation, in addition to an Ag/Ab HIV test (NEW*: good practice statement, indirect data; existing recommendation).” 

On page 14, in the second column, the second bullet should have read, “For persons with long-acting injectable PrEP ARV exposure during the past **6 months**, a diagnostic HIV nucleic acid test (NAT) is recommended at the initial medical evaluation, in addition to an Ag/Ab HIV test (good practice statement, indirect data; existing recommendation).”

On page 15, the fourth sentence in the first paragraph should have read, “Certain experts would include an HIV NAT in nPEP baseline testing, especially if the person has recently taken oral ARVs or had a cabotegravir injection during the past **6 months.**” 

